# The Impact of Alkyl‐Chain Purity on Lipid‐Based Nucleic Acid Delivery Systems – Is the Utilization of Lipid Components with Technical Grade Justified?

**DOI:** 10.1002/cphc.201900480

**Published:** 2019-07-23

**Authors:** Dorota Pawlowska, Christopher Janich, Andreas Langner, Bodo Dobner, Christian Wölk, Gerald Brezesinski

**Affiliations:** ^1^ Max Planck Institute of Colloids and Interfaces, Science Park Potsdam-Golm Am Mühlenberg 1 14476 Potsdam Germany; ^2^ Warsaw University of Technology, Faculty of Chemistry Institute of Biotechnology ul. Noakowskiego 3 00-664 Warsaw Poland; ^3^ Martin Luther University Halle-Wittenberg Institute of Pharmacy, Research Group Biochemical Pharmacy Wolfgang-Langenbeck-Str. 4 06120 Halle (Saale) Germany

**Keywords:** monolayers, lipid dispersions, gene transfection, phase transition, structures

## Abstract

The physicochemical properties and transfection efficacies of two samples of a cationic lipid have been investigated and compared in 2D (monolayers at the air/liquid interface) and 3D (aqueous bulk dispersions) model systems using different techniques. The samples differ only in their chain composition due to the purity of the oleylamine (chain precursor). Lipid **8** (using the oleylamine of technical grade for cost‐efficient synthesis) shows lateral phase separation in the Langmuir layers. However, the amount of attached DNA, determined by IRRAS, is for both samples the same. In 3D systems, lipid **8 p** forms cubic phases, which disappear after addition of DNA. At physiological temperatures, both lipids (alone and in mixture with cholesterol) assemble to lamellar aggregates and exhibit comparable DNA delivery efficiency. This study demonstrates that non‐lamellar structures are not compulsory for high transfection rates. The results legitimate the utilization of oleyl chains of technical grade in the synthesis of cationic transfection lipids.

## 1 Introduction

The main ideas of gene therapy have been well established in recent years. The principle is the use of DNA (or other genetic material) as a drug which is able to alter or supplement a missing or defective gene. However, transfection systems developed thus far suffer from several obstacles.^1^, ^2^ Viruses have been found to be the most efficient DNA vectors. But they may also trigger serious immunological response. Additionally, they are expensive to produce and are, due to their shear particle size, limited in the amount of DNA fragments they can host. The fact that viruses tend to undergo mutations, which can lead to the formation of potentially dangerous virus‐particles with undefined properties, makes them especially problematic for the use in gene therapy. These issues limit their practical utility *in vivo*.^3^, ^4^, ^5^ Thus, the direction of DNA carrier development has been recently focused on non‐viral methods.^6^, ^7^ Cationic lipoplexes (DNA/cationic lipid complexes) are a safe alternative.^8^, ^9^, ^10^, ^11^, ^12^ Generally, they have low immunogenicity,^13^, ^14^ however, each lipid/DNA system has to be individually tested for toxicity.^15^ Also the amount of DNA that can be incorporated is high. In comparison to viral vectors, cationic lipids can be synthesized in large quantities at low cost and easily chemically modified, making them ideal for targeted delivery to specific cells and allowing them to be easily studied and improved. Many synthetic cationic lipids have been already tested with respect to their structure‐transfection efficiency relationship.^16^, ^17^, ^18^ The principal mechanism of lipofection (lipid mediated transfection) relies on electrostatic attractions: cationic lipids bind negatively charged DNA. The overall positive charge of the lipoplex enables its attachment to the negatively charged cell membrane and its up‐take by endocythosis.^19^, ^20^ There are several properties of lipoplexes, however, which have been found important for gene delivery. Apart from the 3D structures of lipoplexes, which are strongly dependent on the chemical and physical properties of the cationic and helper lipids, the efficiency of lipofection is influenced by the amount of incorporated DNA, as well as the charge and pH sensitivity of the lipoplexes. The importance of each of these parameters depends strongly on the studied system. Therefore, it is extremely difficult to predict transfection rates. However, systematic studies of the physical‐chemical properties and the lipid structure/lipofection efficiency correlation may contribute to the optimization of the process.

**Figure 1:**
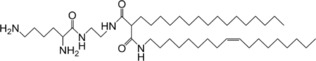
Chemical structure of lipid **8**. Note that the difference between lipid **8 p** and lipid **8** is solely the presence of 25 % of compounds with the same head group structure but different chain patterns.

Recently we reported about a novel class of cytofectines containing a malonic acid diamide backbone.^18^, ^21^, ^22^ The most effective lipids of this class contain at least one oleyl chain. But due to the commercial availability and the low cost, the oleylamine used as precursor was always of technical grade. This choice is a basic requirement to maintain a cheap and simple synthesis. Many groups worldwide use oleyl chains of technical grade to achieve a simple synthesis of cationic lipids with an unsaturated chain,^23^, ^24^, ^25^, ^26^, ^27^ but the effect of the chain composition on the transfection efficiency and structure of the nucleic acid delivery systems was never investigated in detail. Against the background that oleyl or oleoyl chains increase the efficiency,^28^ the synthesis of the lipids should be as simple as possible without decreasing the quality. Hence the question arose, how pronounced is the impact of the alkyl chain purity on the self‐assembly behavior and transfection efficiency. To study this problem we decided to synthesize oleylamine of analytical purity and use it as oleyl chain precursor for the synthesis of lipid **8**, which is the most efficient one among the cytofectines containing the malonic acid diamide backbone.^22^ In this work we compare the physical‐chemical properties and transfection activities of two samples of the same lipid (*N’*‐2‐[(2,6‐diamino‐1‐oxohexyl)amino]ethyl‐2‐hexadecyl‐*N*‐[(*9Z*)‐octadec‐9‐enyl]propane diamide, Figure 1) with different qualities of purity. One is of analytical pure grade (named lipid **8 p**) and the second one is of technical grade (named lipid **8**). Lipid **8** has been already described,^29^, ^30^ and we will therefore focus on the physical‐chemical behavior of lipid **8 p** and the differences between the two samples.

Since it is assumed that cationic lipids with unsaturated chains exhibit enhanced transfection efficacy,^22^, ^28^ the main aim of this publication is to screen the impact of chain purity on the phase behavior and transfection rates. We will clearly demonstrate that 15 % of saturated cationic lipids in the cost‐efficient technical mixture are not at all harmful for lipofection purposes in three different cell lines. Nevertheless, structural differences have been found between both lipid formulations. The physical‐chemical properties and the interaction with DNA of both samples have been studied in monolayers (2D systems) using a Langmuir trough in combination with various analytical methods (Infrared Reflection Absorption Spectroscopy (IRRAS) and Grazing Incidence X‐ray Diffraction (GIXD)) as well as in bulk (3D systems) using Small‐ and Wide‐Angle X‐Ray Scattering (SAXS and WAXS) and Differential Scanning Calorimetry (DSC). The *in vitro* transfection properties have been studied with an enhanced green fluorescent protein (eGFP)‐assay, and the cytotoxic properties with the alamarBlue®‐assay.

## Experimental Details

### General

The synthesis of *N'*‐2‐[(2,6‐diamino‐1‐oxohexyl)amino]ethyl‐2‐hexadecyl‐*N*‐[(9*Z*)‐octadec‐9‐enyl]propane diamide is described in the Supporting Information. All materials and reagents were purchased from Sigma‐Aldrich Co Ltd. unless stated otherwise. All solvents were analytically pure and dried before use. Thin layer chromatography was carried out on aluminum plates pre‐coated with silica gel 60 F254 (Merck, Darmstadt, Germany) and developed with bromothymol blue dip. For column chromatography under normal pressure, silica gel 60 (0.063–0.200 mm) was used. Mass spectrometry analyses were performed with a Finnigan MAT 710 C (Thermoseparation Products, San Jose (Ca), USA) for ESI‐MS, and with a LTQ‐Orbitrap‐mass‐spectrometer (Thermo Fisher Scientific, Bremen, Germany) for HR‐MS. ^1^H‐NMR and ^13^C‐NMR spectra were recorded on a Varian Gemini 2000 and a Varian Inova 500 using CDCl_3_ as a solvent. Elemental analyses were performed with a CHNS‐932 (Leco‐Corporation, St. Joseph (MI), USA). Milli‐Q water with a specific resistance of 18.2 MΩcm was produced with a Milli‐Q Advantage A10 pure water system (Millipore, Billerica (MA), USA). Sodium salt of calf thymus deoxyribonucleic acid (D1501) (ctDNA) was purchased from Sigma‐Aldrich and used in a concentration of 0.1 mM (refers to a monomer containing one charge per phosphate moiety, ∼370 g/mol) for monolayer experiments and 2 mg/mL for bulk experiments. For all experiments at pH 10 and pH 4 carbonate (5 mM) and citric (5 mM) buffers were used, respectively. All other chemicals were of analytical grade and used without further purification.

### Monolayer Experiments

The cationic lipid in chloroform (0.5 mM) was spread onto the buffer subphase with a micro‐syringe and left for 10 minutes before compression for complete solvent evaporation. For DNA experiments, 0.1 mM ctDNA in the relevant buffer was used as subphase. The lipid monolayer was spread onto the DNA containing subphase and left in the uncompressed state for one hour to equilibrate DNA adsorption. Two different buffers were used for the experiments: acidic citric buffer of pH 4 and basic carbonate buffer of pH 10.

***Film Balance Measurements***. The pressure/area (π/A) isotherms were measured on a computer‐interfaced Langmuir trough (R&K, Potsdam, Germany) equipped with a Wilhelmy type surface pressure microbalance. The films were compressed at a rate of 2.1 Å^2^/(molecule ⋅ min). All measurements were performed at a constant subphase temperature (indicated) with an accuracy of 0.1 °C.^31^

#### Infrared Reflection‐Absorption Spectroscopy (IRRAS)

Infrared reflection‐absorption spectra (IRRA spectra) were recorded on a Vertex 70 FT‐IR spectrometer (Bruker, Ettlingen, Germany) equipped with Mercury Cadmium Telluride (MCT) detector cooled with liquid nitrogen. The IR beam was polarized by a KRS‐5 wire grid polarizer in the plane of incidence (p) and perpendicular to this plane (s). Spectra were recorded with a resolution of 8 cm^−1^ and a scanning velocity of 20 kHz. For each single beam spectrum 200 scans of s‐polarized light and 400 scans for p‐polarized light were collected. The angle of incidence normal to the surface was set to 40°. The Langmuir trough used for the IRRAS measurements has two movable barriers allowing symmetric compression of the monolayer.^32^, ^33^, ^34^, ^35^, ^36^

#### Grazing Incidence X‐ray Diffraction (GIXD)

All measurements were performed using the liquid surface diffractometer at the undulator beamline BW1 (HASYLAB, DESY, Hamburg, Germany). The Langmuir trough was located in a thermostated, hermetically closed container flushed with He. The synchrotron beam was monochromated by a beryllium (002) crystal to a wavelength of 1.304 Å. The incidence angle at the liquid surface was 0.11°, which is ∼85 % of the critical angle for total external reflection from water at this X‐ray energy. A MYTHEN detector system (PSI, Villigen, Switzerland) was used to record the intensity of the diffracted beam as a function of the vertical scattering vector component (*Q_z_*≈(2π/*λ*)sin*α_f_*) and the horizontal scattering vector component (*Q_xy_*≈*(*4π*/λ)*sin*(2θ/2*)), where *α_f_* is the vertical and 2θ the horizontal scattering angle. The horizontal resolution (0.008 Å^−1^) was determined by a Soller collimator (JJ X‐RAY, Denmark) located in front of the detector. The intensities were corrected for polarization, effective area, and Lorentz factor. Model peaks, taken as Lorentzian in the in‐plane direction (Bragg peak, *Q_xy_*) and as Gaussian in the out‐of‐plane direction (Bragg rod, *Q_z_*), were fitted to the corrected intensities. The *Q_xy_* and *Q_z_* values were used to calculate lattice unit cell parameters, unit cell distortion (*d*), chain tilt angle (*t*) and chain cross‐sectional area (*A_0_*). The finite size *L_xy_* of crystalline domains in a monolayer can be determined with the Scherrer formula *L_xy_*=0.88 ⋅ (2π/*ΔQ_xy_*) where *ΔQ_xy_* is the full‐width at half‐maximum (fwhm) of the Lorenzian peak corrected with the detector resolution. The thickness of the scattering unit (length of a chain contributing to the signal) can be calculated using *L_z_*=0.88 ⋅ (2π/*ΔQ_z_*), with *ΔQ_z_* being the fwhm of the Gaussian peak.^37^, ^38^, ^39^, ^40^, ^41^

### Bulk Experiments

#### Synchrotron Small‐ and Wide‐Angle X‐ray Scattering Experiments (SAXS/WAXS)

SAXS enables the determination of long‐range organization of fully hydrated lipids in bulk. WAXS gives information about the layer in‐plane structures reflecting the lipid packing. SAXS/WAXS experiments were performed at the Soft Condensed Matter beamline A2 (HASYLAB, DESY, Hamburg, Germany). Aqueous dispersions of the lipids or their mixtures with cholesterol were prepared (20 weight‐% lipid), heated up to 80 °C, vortexed and placed into glass capillaries. The lipid/cholesterol mixtures used for transfection experiments have been prepared in chloroform. After drying the sample, the mixed film was re‐dispersed with an aqueous solution containing the appropriate amount of model DNA to obtain the desired N/P ratio and placed into glass capillaries. SAXS and WAXS patterns of samples were measured simultaneously by a MAR CCD detector (Evanston, Illinois, USA) and a linear detector with delay‐line, respectively. The incoming beam had a wavelength of 0.15 nm. Exposure time was 30 s. All data were recorded in the temperature range between 20 °C and 70 °C in 5 °C steps. The Bragg peak maxima were determined by Lorenzian fit. The obtained *s*‐values were translated into the spacing of lattice planes, *d*, using *s*=1/*d*.^30^, ^42^, ^43^, ^44^

#### Differential Scanning Calorimetry (DSC)

The corresponding lipid was dispersed to a concentration of 1 mg/mL using different media (5 mM carbonate buffer (pH 10, Na_2_CO_3_/NaHCO_3_), 5 mM citric buffer (pH 4, C_6_H_8_O_7_/Na_3_C_6_H_5_O_7_), water). Mixtures with cholesterol were prepared in a final concentration of 3 mM. Before film rehydration, the lipids were dissolved in CHCl_3_/methanol (7 : 3 v:v), the organic solvent was evaporated under a nitrogen stream, and the film was dried for 12 hours in vacuo. The hydrated samples were heated two times to 80 °C and vortexed followed by 20 min sonication at 60 °C. Finally, the samples were degassed for 15 min.

The DSC measurements were performed on a MicroCal VP‐DSC (MicroCal Inc. Northampton, MA, USA). The heating rate was 60 °C/h in the temperature range between 2 °C and 95 °C. Each heating and cooling scan was repeated to confirm reproducibility, and the first scan was abolished. The reference cell was filled with the pure solvent. The buffer‐buffer baseline was subtracted from the thermograms of the samples, and the DSC scans were evaluated using MicroCal Origin 8.0 software.

### Biological Experiments

The transfection experiments were carried out on adherent growing human epithelial lung carcinoma cells (A549), human cervix adenocarcinoma epithelial cells (HeLa), and pig kidney epithelial cells (LLC‐PK1). *In vitro* transfection properties were studied with the eGFP‐assay and the cytotoxic properties were checked with the alamarBlue®‐assay. The exact procedures and data evaluation methods are described in Supporting Information.

## 2 Results

### 2.1 Synthesis and Purity of the Compounds

Since pure (*9Z*)‐octadec‐9‐en‐1‐ylamine (oleylamine) is not commercially available, it had to be synthesized from pure (*9Z*)‐octadec‐9‐enoic acid (oleic acid) in multiple time‐ and cost‐intensive steps.

**Figure 2:**
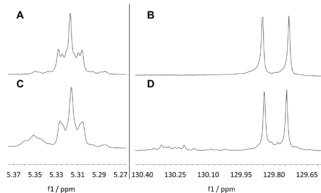
Details of the ^1^H‐ and ^13^C‐NMR spectra of oleylamine pure and oleylamine technical grade in CDCl_3_. Left panel: ^1^H‐NMR spectra showing the signals of the olefinic protones of oleylamine pure (A) and oleylamine technical grade (C). Right panel: ^13^C‐NMR spectra showing the signals of the olefinic carbons of oleylamine pure (B) and oleylamine technical grade (D).

Technical oleylamine has been used as a relatively inexpensive alternative by many groups for lipid synthesis.^21^, ^22^, ^23^, ^24^, ^26^, ^27^ GC‐MS analysis (see Supporting Information, Figure S2) shows that this multicomponent system contains only 75 % oleylamine. The other compounds are amines with saturated chains (1.7 % tetradecylamine, 5.9 % hexadecylamine, 0.8 % heptadecylamine, 6.2 % octadecylamine) and other unsaturated chains (5.5 % (*9Z*)‐hexadec‐9‐en‐1‐ylamine, 1.5 % (*9Z*)‐heptadec‐9‐en‐1‐ylamine), and 3.4 % unidentified compounds. However, this product is a primary amine to 98 %. Figure 2 shows details of the ^1^H‐NMR spectrum with the signals of the olefinic protones. The presence of both *trans* olefinic protones (m, f1 from 5.34–5.37 ppm) and *cis* olefinic protones (m, f1 from 5.29–5.34 ppm) is clearly visible. Integration of the signals as well as Gaussian fit analysis indicates a *trans* olefinic fraction of ∼20 % and a *cis* olefinic fraction of ∼80 %. Also, the ^13^C‐NMR spectrum shows the presence of *trans* double bonds (Figure 2: signals between 130.1 and 130.4 ppm). A comparable *cis*/*trans*‐ratio was also described for oleyl chain containing gemini surfactants for gene transfer by Johnsson et al.^45^ using commercially available oleyl alcohol. Here it is important to note that *trans* isomers have been described as better transfectants than *cis* isomers.^28^, ^46^

For the synthesis of lipid **8 p**, pure oleylamine synthesized from oleic acid (purity grade≥99 %) was used. The synthesis is shown in the supplementary information (Figure S1). The applied synthesis is faster and the reaction conditions are milder than the synthesis strategies described in literature.^47^ The yield is comparable to that in literature. The ^1^H‐ and ^13^C‐NMR‐spectra clearly demonstrate the presence of only *cis* double bonds (Figure 2 A/B), and GC‐MS analyses (see Supporting Information, Figure S3) show the absence of other derivatives. To assure that the following reaction steps did not affect the configuration of the double bond, the NMR‐data of the final lipid **8 p** (see Supporting Information for ^1^H‐NMR‐, ^13^C‐NMR‐, and HSQC‐spectra, Figure S4) have been compared with those of the pure oleylamine. The spectra show equivalent signals in the olefinic region, and no signals attributable to *trans* double bonds appeared.

### 2.2 Monolayer Experiments (2D Systems)

The monolayer experiments were performed to compare the physical‐chemical properties and the ability of the cationic lipid to bind DNA in absence of a co‐lipid and to screen for de‐mixing effects.

#### 2.2.1 Monolayer Experiments on Buffer Solutions

##### 2.2.1.1 Pressure‐Area Isotherms

**Figure 3:**
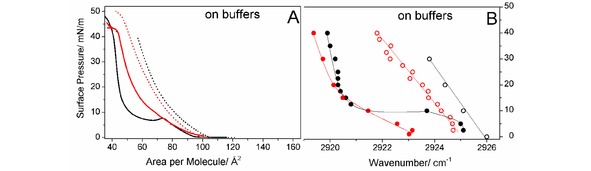
Pressure/area isotherms (A) and positions of the asymmetric CH_2_ stretching vibration band (B) along the compression isotherm at 20 °C of lipid **8 p** (black) and lipid **8** (red) on buffers: citric buffer pH 4 (A: dashed line, B: empty circles) and carbonate buffer pH 10 (A: solid line; B: filled circles). The lines in B are only for guiding the eye.

The π/A isotherms of lipids **8** and **8 p** on the pH 10 buffer at 20 °C are presented in Figure 3A. Lipid **8 p** undergoes a first‐order phase transition from the liquid‐expanded (LE) to a liquid‐condensed (LC) state represented by the characteristic plateau region.^31^ Since the transition is very well defined, the isotherms have been measured at different temperatures (Figure S6) to extract thermodynamical parameters of the phase transition (see Supporting Information), resulting in a critical temperature (T_c_) of 45.4 °C. The beginning of the transition region of lipid **8 p** at pH 10 is characterized by a small hump typical for an over‐compression needed to activate nucleation. At pH 10, the molecules are considered to be deprotonated (uncharged). The charge is introduced by the possible protonation of the two primary amine groups in the head group of the molecules at lower pH values (pH 4). This leads to the expansion of the monolayer due to electrostatic repulsion (Figure 3A). The same scenario can be observed for lipid **8**. However, the clear first‐order phase transition at pH 10 cannot be detected in the isotherm (Figure 3A). The reason for the missing plateau region must be the multi‐component character of lipid **8** resulting from the variation in the alkyl chain composition. Instead, a continuous change of molecular areas from values characteristic for an expanded phase to values typical for a condensed phase can be seen on compression. BAM images (Supporting Information, Figure S7) show the coexistence of different phases. Even at pH 4, small condensed domains floating in the expanded phase of lipid **8** can be seen.

##### 2.2.1.2 IRRAS

The lipid phase state along the isotherms was checked by the position of the asymmetric CH_2_ *‐*stretching vibration band. The LE phase is characterized by positions of the symmetric and asymmetric CH_2_ *‐*stretching vibration bands above 2854 cm^−1^ and 2924 cm^−1^, respectively, while for the LC phase lower values (<2850 cm^−1^ and <2920 cm^−1^, respectively) are observed.^32^, ^33^, ^35^, ^36^, ^48^

The ν_as_(CH_2_) band positions of lipids **8 p** and **8** on different buffers (pH 10 and pH 4) are plotted as a function of the lateral surface pressure at 20 °C in Figure 3B. At pH 4, lipid **8 p** is clearly in the LE phase at all lateral pressures investigated due to electrostatic repulsion between protonated head groups. For lipid **8**, the band is located at slightly smaller wavenumbers in comparison to lipid **8 p**, which is the result of the presence of ∼15 % of saturated chains in the multi‐component system, which remain in the condensed state even at low pH values (condensed domains shown by BAM in Figure S7). Therefore, lipid **8** exhibits a smooth LE/LC transition at basic pH because a certain percentage of the molecules form the condensed phase already at low pressure whereas others are in the disordered state. In contrast, lipid **8 p** exhibits the classical first‐order phase transition in the isotherm which is characterized by a sharp decrease of the wavenumber of the ν_as_(CH_2_) band. The phase transition pressure amounts to ∼10 mN/m, which is not exactly the equilibrium value observed in the isotherm (∼8 mN/m) at the same temperature but corresponds rather to the over‐compressed state (hump in the isotherm) due to the experimental procedure (experiments are performed at a fixed pressure).

##### 2.2.1.3 GIXD

**Figure 4:**
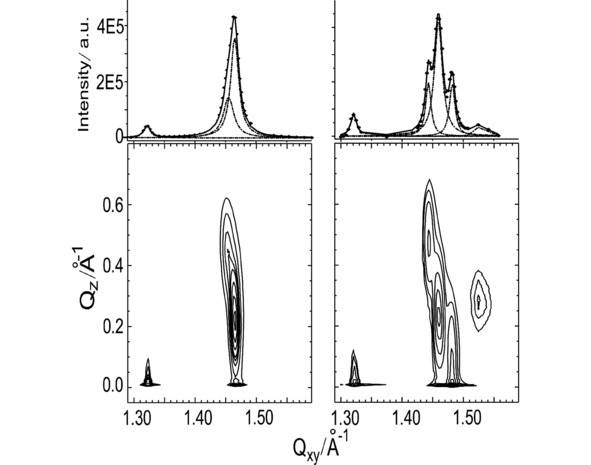
Bragg peaks (top panel) and contour plots of the scattered intensity as a function of the in‐plane scattering vector component *Q_xy_* and the out‐of‐plane scattering vector component *Q_z_* (bottom panel) of lipid **8 p** (left) and lipid **8** (right) monolayers on buffer, pH 10 or pH 8, correspondingly, at 10 mN/m and 5 °C.

GIXD enables the determination of the lattice structure in LC phases. Lipid **8 p** and lipid **8** were studied at pH 4 and pH 10 at 5 °C and different surface pressures. At pH 4, lipid **8 p** is in the LE phase (in agreement with the isotherm and IRRAS data (Figure S5)) and no diffraction pattern is observed (experiments have been performed only up to 30 mN/m because of layer stability problems). In contrast, monolayers of lipid **8** exhibit already at low pressure a very weak diffraction pattern at pH 4 even if the isotherm has the typical shape of an expanded layer. The molecular areas are only slightly smaller compared with lipid **8 p** but the corresponding ν_as_(CH_2_) are drastically shifted by 3 wavenumbers to smaller values (Figure S5). Obviously, the 15 % of lipids with saturated chains are responsible for these observations in perfect agreement with the condensed domains observed in BAM (Figure S7).^52^ Since the head groups are uncharged at high pH values (pH>8), a condensed phase is observed at lower surface pressures. Figure 4 shows intensity contour plots of lipid **8 p** and lipid **8** at pH 10 or pH 8, correspondingly. The *Q_xy_* and *Q_z_* values of the Bragg peaks, as well as the distortion *d,* the tilt *t* and the cross‐sectional area *A_0_* of lipids **8 p** and **8** are summarized in table S1 (Supporting Information).

The contour plot of lipid **8 p** presents three well resolved diffraction peaks. The peak located at *Q_xy_*=1.322 Å^−1^ (corresponding to a *d*‐value of 4.76 Å) and *Q_z_*=0 characterizes the formation of a hydrogen bond network which can be formed between the amide groups (NH as proton donor and C=O as proton acceptor). This Bragg peak does not change the position with increasing surface pressure, since the hydrogen bond length is well defined and fixed for a given type of interaction. The two other peaks can be assigned to a lattice formed by the lipid chains. The corresponding Bragg rods are located above the horizon (*Q_z_* ^*nd*^=2 ⋅ *Q_z_* ^*d*^ >0). Such a diffraction pattern is typical for a rectangular unit cell with NNN (next‐nearest neighbor) tilt of the chains (such a phase is typically called Ov). Since the degenerated Bragg peak is located at higher *Q_xy_* value than the non‐degenerate peak, the unit cell is distorted in NNN direction.^49^ Upon compression (from 5 to 30 mN/m), the two Bragg peaks change their positions in *Q_xy_*. This indicates a change in the distortion from NNN to NN (nearest neighbor). However, compression leads only to marginal changes of the chain tilt angle (17° at 5 mN/m to 14° at 30 mN/m, see Table S1, Supporting Information). The theoretical tilting phase transition pressure can be calculated by assuming that the molecular in‐plane area (*A_xy_*) depends linearly on the surface pressure and the chain cross‐sectional area, *A_0_*, is constant. The extrapolation of 1/cos(t) versus *π* towards zero tilt angle (1/cos(t)=1) yields the transition pressure to the non‐tilted state.^54^ For both lipids, the determined transition pressures are extremely high (Figure S8), showing that the non‐tilted state cannot be reached by compression due to the lattice immobilization by hydrogen bonds. Lipid **8 p** has a chain cross‐sectional area of ∼20.4 Å^2^, which is typical for freely rotating chains but the unit cell is distorted. The H‐bonding network, formed by linking head groups of neighboring molecules, leads to the observed distortion but allows the chain rotation. Both observations are in perfect agreement with the determined marginal changes of the chain tilt. The fwhm of the Bragg rods can be directly translated into the length of the scattering unit *L_z_. L_z_* of lipid **8 p** amounts to ∼21 Å. This value can be compared with the theoretical length of the alkyl chains of lipid **8 p** forming the hydrophobic part of the amphiphilic layer (1.26 Å per C−C in an *all‐trans* conformation+1.5 Å for the terminating CH_3_ group)^50^, ^51^ of ∼22 Å, showing that the whole chain is contributing to the scattering.

GIXD of lipid **8** shows a diffraction pattern originating from the superposition of two contributions of the multi‐component system. Five well resolved peaks can be seen in the contour plot presented in Figure 4. Two pairs of Bragg peaks belong to different lipid **8** chain lattices. One of them (*Q_xy_*=1.443 Å^−1^, *Q_z_*=0.498 Å^−1^ and *Q_xy_*=1.459 Å^−1^, *Q_z_*=0.249 Å^−1^) is very similar to the one observed for lipid **8 p** with only slightly different values giving rise to a slightly larger chain tilt angle (*t*=19°, NNN) due to a partial miscibility with the other components of the mixture. The cross‐sectional area per chain is not affected and equals 20.4 Å^2^. The other two Bragg peaks (*Q_xy_*=1.481 Å^−1^, *Q_z_*=0 Å^−1^ and *Q_xy_*=1.528 Å^−1^, *Q_z_*=0.263 Å^−1^) belong most probably to a chain lattice of the compounds with saturated chains. This lattice has a much smaller cross‐sectional chain area (*A_0_*=19.5 Å^2^) and tilt angle (*t*=11.8°, NN) due to the tighter packing of the saturated chains. The fifth peak at *Q_xy_*=1.321 and *Q_z_*=0 corresponds again to the hydrogen bond network and is at the same position as that found in the lipid **8 p** monolayer.

#### 2.2.2 Monolayer Experiments on DNA Solutions

##### 2.2.2.1 Pressure‐Area Isotherms

**Figure 5:**
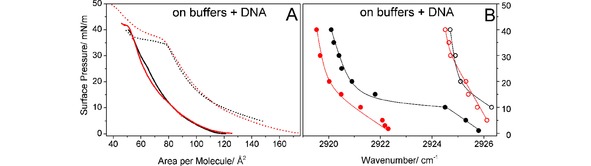
Pressure/area isotherms (A) and the positions of ν_as_ (CH_2_) band along the compression isotherm (B) at 20 °C of lipid **8 p** (black) and lipid **8** (red) on 0.1 mM solution of ctDNA in buffers: citric buffer, pH 4 (A: dashed line, B: empty circles) and carbonate buffer, pH 10 (A: solid line; B: filled circles). The lines in B are only for guiding the eye.

On DNA containing subphases, the isotherms are characterized by an expansion effect (Figure 5A). The reason for the pH dependent expansion could be electrostatic interaction between cationic lipids and anionic DNA as well as partial penetration of DNA molecules into the lipid monolayer. The apparent areas per molecule (only the known number of lipids at the surface can be used for the calculation of molecular areas) are much larger at pH 4 than at basic pH. At pH 10, both lipid layers can be compressed to quite small areas per molecule (∼55 Å^2^). However, no obvious phase transition can be seen in the isotherms. There is also almost no difference in the strongly expanded isotherms of lipid **8 p** and lipid **8** after DNA coupling at pH 4. The apparent plateaus above 35 mN/m do not indicate first‐order phase transitions (see IRRAS results). Concluding, the interaction of both lipids with DNA leads to very similar compression isotherms.

##### 2.2.2.2 IRRAS

At pH 4, the electrostatic interaction between the protonated lipid head groups and the negatively charged DNA leads to the expansion of both monolayers. The wavenumbers (Figure 5B) indicate a completely fluid state of the alkyl chains at all pressures investigated. The influence of the components with saturated chains in lipid **8** on the wavenumbers cannot be seen anymore, indicating that all components in the multi‐component mixture are now in the fluid state in contrast to the observation on the pure buffer. At pH 10, lipid **8 p** shows the same wavenumber dependence as observed on the pure buffer. In contrast to the isotherms (Figure 5A), the first‐order phase transition can be clearly seen at ∼10 mN/m. This proves that there is no interaction between the uncharged lipids and the charged DNA. The expanded isotherms indicate only that at low pressure (low lipid density at the air/buffer interface) a small amount of DNA molecules can penetrate into the liquid lipid film, but will be squeezed‐out upon compression. In this case, the counterions from the buffer solution screen the DNA charges and allow the DNA adsorption at the air/liquid interface. The same is valid for lipid **8** with slightly lower wavenumbers and the more continuous transition due to the presence of components with saturated chains.

##### 2.2.2.3 Quantification of Bound DNA

Since DNA is the only compound exhibiting phosphate groups, the relative amount of DNA attached to the monolayer can be estimated by the determination of the intensity of typical symmetric, ν_s_ (PO_2_), and asymmetric, ν_as_ (PO_2_), phosphate diester bands at around 1082 cm^−1^ and 1238 cm^−1^, respectively (spectra in Figure S9). The presence of DNA can also be deduced from the DNA backbone band at 970 cm^−1^.^52^, ^53^, ^54^ To check a possible influence of the orientation of DNA molecules on the signal intensity, the dichroic ratio (ΔR) was calculated with ΔR=A_p_/A_s_ where A_p_ and A_s_ are the maxima of reflectance absorbance (RA) obtained with p‐ and s‐polarized light, respectively. No changes of ΔR have been observed.

**Figure 6:**
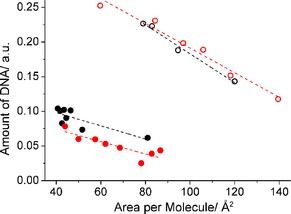
Amount of DNA bound to lipid **8 p** (black) and lipid **8** (red) monolayers spread on 0.1 mM DNA in citric buffer, pH 4 (empty circles) and carbonate buffer pH 10 (filled circles) along the compression isotherm at 20 °C.

In Figure 6, the phosphate band intensity as a measure of the amount of DNA bound to lipid **8 p** and lipid **8** monolayers at pH 4 and pH 10 is plotted versus the area per molecule. It is worth to remember that the areas from the isotherms are only apparent ones. Therefore, the curve progression might be wrong, but the difference between pH 4 and 10 is non‐ambiguous. It can be concluded that decreasing pH (increasing protonation degree) increases the amount of attached DNA. At pH 10, the signal intensity in the phosphate region is extremely small leading to the conclusion that only a small amount of DNA is attached to or penetrated into the lipid monolayers at high pH values. At pH 4, a considerable amount of DNA is bound to the lipid monolayers. There is no difference between the two samples (pure and mixed systems) concerning the amount of attached DNA. Compression of the lipid layers leads to an increase in the charge density and more DNA is attracted.

### 2.3 Bulk Experiments (3D Systems)

The lipids **8 p** and **8** as well as their mixtures with cholesterol and DNA have been examined in bulk by DSC and SAXS/WAXS experiments. The mixing ratio of lipid to cholesterol of 1 : 1 was chosen based on the fact that this is the best transfecting mixture in a series of systematically designed transfection complexes with different ratios.

#### 2.3.1 Pure Lipids

##### 2.3.1.1 DSC

**Figure 7:**
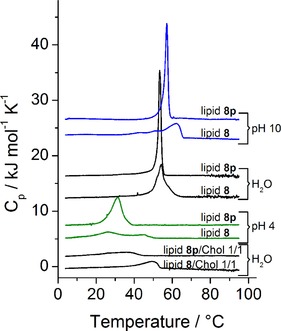
DSC heating scans of aqueous dispersions of lipid **8 p** and lipid **8** (c=1 mg ⋅ mL^−1^) and their mixtures with cholesterol (n/n 1/1) (c=3 mM) (media: carbonate buffer 10 mM, pH 10, water, citric buffer 5 mM, pH 4). The heating rate was 60 K ⋅ h^−1^. Curves are shifted vertically for clarity.

Phase transitions in aqueous dispersions have been investigated by means of DSC (Figure 7). By comparing the peak shape of the heating curves of lipid **8** and lipid **8 p** it is obvious that the ones of lipid **8** are broader and less intense at all three investigated pH values, a typical phenomenon for lipid mixtures.^55^, ^56^ The corresponding *T_m_*, ▵*H*, ▵*S*, and the fwhm (full‐width at half‐maximum) values are summarized in table S2. The two buffer systems exhibit defined pH values, the aqueous lipid dispersions show pH values between 7 and 8 due to the addition of the basic lipids. The *T_m_* values of both systems, lipid **8 p** and lipid **8**, decrease with decreasing pH. Furthermore, both lipid systems exhibit the lowest ▵*H* and ▵*S* values as well as the largest fwhm values in citric buffer (pH 4). This behavior is in accordance with the increased protonation degree (increasing electrostatic repulsion) leading to the destabilization of the gel phase.^57^ At the same pH, the *T_m_* values of lipid **8** are higher compared to those of lipid **8 p** due to the presence of 15 % amines with saturated chains. Detailed analysis of the DSC curves of lipid **8** shows additional peaks/shoulders due to de‐mixing processes in the multi‐component sample.^55^, ^56^

##### 2.3.1.2 SAXS/WAXS

**Figure 8:**
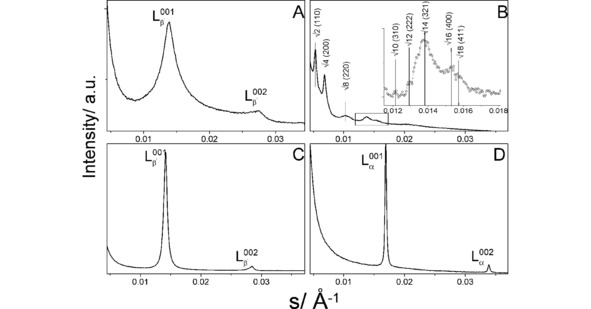
SAXS pattern of lipid **8 p**: A ‐ in water at 30 °C (below the main phase transition); B – in water at 70 °C (above the main phase transition). The Bragg peaks belonging to the cubic phase are indexed and indicated (the √6 peak is missing); C – in pH 10 buffer at 30 °C (below the main phase transition); D – in pH 10 buffer at 70 °C (above the main phase transition).

Lipid **8** was intensively characterized by means of SAXS and WAXS in previous research.^30^ The SAXS patterns of dispersions of lipid **8 p** (20 wt %) in water and carbonate buffer (pH 10) below and above the main phase transition temperatures are shown in Figure 8. At both pH values, the gel phase is characterized by two Bragg peaks with a *s_1_:s_2_* ratio of 1 : 2 indicating a multilamellar structure. The gel phase WAXS pattern of lipid **8 p** (Figure S12B) in carbonate buffer is characterized by two Bragg peaks corresponding to an orthorhombic lipid chain lattice (*s_1_*=2.415 nm^−1^ and *s_2_*=2.469 nm^−1^ at 30 °C) perpendicular to the chain axis typical for lamellar gel phases with tilted chains, L_β’_.^58^ The cross‐sectional area of the chains amounts to *A_0_*=19.5 Å^2^, and is therefore smaller than the one determined by GIXD in monolayers (*A_0_*=20.4 Å^2^). A different packing of the lipid in bilayers and monolayers could be already expected based on the determined inequality of T_m_ (pH 10: 57.1 °C) and T_c_ (pH 10: 45.4 °C). Above *T_m_*, only a broad halo characteristic for fluid chains can be seen. Therefore, lipid **8 p** shows the typical transition from a lamellar gel phase (L_β’_) to a lamellar liquid‐crystalline phase (L_α_) at pH 10. This transition is accompanied with a shift of the Bragg peaks in the SAXS pattern to larger *s*‐values (from *d*=70.9 Å at 30 °C to *d*=59.5 Å at 70 °C) (Figure S12A). The observed decrease of ∼11 Å in the bilayer thickness can be solely explained by the melting of the chains keeping the water layer between the lipid bilayers almost constant.

The diffraction peaks from the gel phase observed in water are broader indicating smaller correlation length between the bilayers. The gel phases in water of both lipids **8 p** and **8** are characterized by the same *d*‐values. The *d*‐value of lipid **8 p** decreases slightly with increasing temperature from 72.5 Å at 30 °C to 71.2 Å at 55 °C **(**Figure S10A). The gel phase WAXS patterns of lipid **8 p** (Figure S12B) in water are again characterized by two Bragg peaks (L_β’_). The additional weak Bragg peak at *s*=2.15 nm^−1^ (*d*=0.465 nm) is typical for hydrogen bonds which can be formed between amine and carbonyl oxygen in the hydrophilic head group region. The packing density is lower in the lipid **8** bilayers (larger *A_0_* values) compared to the pure lipid **8 p**. In both cases, *A_0_* increases slightly with increasing temperature. The melting in water is connected with the appearance of a lamellar L_α_ phase (*d*=63.6 Å at 60 °C), but several other Bragg peaks in a ratio characteristic for a cubic phase appear additionally (Figure 8B). The cubic phase has been identified as body‐centered cubic with Im3 m symmetry. Figure S13 presents the *s* values as function of (h^2^+k^2^+l^2^)^1/2^ with h, k, l as the corresponding Miller indices of the Bragg peaks. The dependence is linear and the extrapolation goes through zero what proves the correctness of the structure identification.^59^ The calculated cubic unit cell parameter, a=1/slope of the linear fit, amounts to 265 Å. At 70 °C, only the Q_α_ phase is present in the system.

At pH 4, a similar behavior has been observed. A reasonable scenario is therefore the formation of a lamellar L_α_ phase at high pH in the non‐protonated state, which transforms into the Q_α_ phase at elevated temperatures, and a cubic phase at low pH in the fully protonated state.

#### 2.3.2 Lipid/Cholesterol 1 : 1 Mixtures

##### 2.3.2.1 DSC

Additionally, mixtures of lipid **8 p** and lipid **8** with cholesterol (50 mol %) have been investigated in water. The addition of cholesterol decreases *T_m_* and increases the fwhm (lipid **8 p**/chol 1/1: *T_m_*=35.2 °C, fwhm=18.6 °C; lipid **8**/Chol 1/1: *T_m_*=49.3 °C, fwhm=9.0 °C) (see Table S2). This phenomenon is also described for other phospholipid/cholesterol mixtures.^60^ But in contrast to phospholipid/cholesterol mixtures, in which the main transition disappears at 40–60 % cholesterol, we clearly see a phase transition in the 1/1 mixtures. This could indicate that only a small part of cholesterol is incorporated into the lipid gel phase. The lipid **8**/chol mixture undergoes the phase transition between 40 and 55 °C, lipid **8 p**/chol between 25 and 45 °C.

##### 2.3.2.2 SAXS/WAXS

SAXS and WAXS patterns of the mixture lipid **8 p**/chol 1/1 are shown in Figure S14. Below the main phase transition, a lamellar structure is observed. The Bragg peak at s_001_=0.014 Å^−1^ shows that the incorporation of cholesterol leads only to a small decrease of the *d*‐value by ∼1 Å. Obviously, only a little cholesterol is incorporated into the lipid gel phase, a large amount of cholesterol is phase separated as anhydrous crystals (L_c_) indicated by a Bragg peak at s=0.029 Å^−1^. These anhydrous crystals show a low enthalpy transition at 37 °C in water, which is much below the melting point around 148 °C, in accordance with literature data.^61^, ^62^ The lipid **8 p**/chol mixture forms the L_α_ phase with similar *d*‐values as lipid **8 p** alone in coexistence with the body‐centered cubic phase above the main phase transition temperature (the broad transition range is finished at ∼45 °C). The small percentage of incorporated cholesterol lowers the *T_m_* value by changing the in‐plane packing in the gel phase without noticeable change of the interlamellar repeat distance *d*. The appearance of two coexisting phases (L_α_ and Q_α_) just above the main phase transition is not influenced by the incorporated cholesterol. At higher temperatures (50 °C), no Bragg peaks of cholesterol crystals can be seen in the diffraction patterns indicating that now all cholesterol is incorporated into the two liquid‐crystalline phases L_α_ and Q_α_. The cooling DSC scan (Figure S15) shows a larger hysteresis with two exothermic transitions. The first one at higher temperature (35 °C) with the much lower enthalpy change must be connected with the Q_α_–L_α_ transition. The second transition with the much larger *ΔH* value at 28 °C is then the transition into the gel state connected with the formation of phase‐separated crystalline cholesterol.

For lipid **8**/chol, only one SAXS measurement at 20 °C has been described^30^ showing that the gel phase structure is also unchanged, except a small decrease of the *d* value by ∼1.5 Å, and a large part of cholesterol is phase‐separated. WAXS experiments to detect a possible in‐plane phase separation between the saturated and unsaturated components have not been performed.

#### 2.3.3 Lipid/Cholesterol 1 : 1 Mixtures Complexed with DNA

##### 2.3.3.1 SAXS/WAXS and DSC

**Figure 9:**
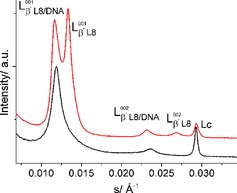
Comparison of SAXS patterns of water dispersions of lipid **8 p**/chol/DNA (black) and lipid **8**/chol/DNA (red) complexes at 20 °C. The diffraction pattern of the lipid **8**/chol/DNA mixture shows phase separation into two phases (L_β’ L8/DNA_) and (L_β’ L8_), whereas the lipid **8 p**/chol/DNA mixture exhibits one homogeneous phase filled with DNA. In both cases, phase separated cholesterol can be observed (L_c_).

Figure S16 shows SAXS patterns of lipid **8 p**/chol (1 : 1) mixed with DNA (N/P ratio 3 : 1, where N refers to the amino groups of the lipid, which can be protonated, and P to the negatively charged phosphate groups of DNA). At lower temperatures (gel phase) three SAXS diffraction peaks can be observed. The Bragg peak at s=0.029 Å^−1^ belongs to the phase‐separated crystalline anhydrous cholesterol. The two other peaks (s_001_=0.0118 Å^−1^ and s_002_=0.0236 Å^−1^ at 20 °C) are in a ratio of 1 : 2 and belong to the lamellar phase of the lipid **8 p**/chol/DNA complex with an increased *d* value (84.7 Å). It has been reported that incorporated DNA can exhibit a one‐dimensional periodicity between aligned strands located between the lipid layers,^63^, ^64^, ^65^ but in the present case no additional DNA peak was observed. The *d* values of the lipoplex are approximately 12 Å larger compared to the *d* values of the gel phases of the pure lipid **8 p** and the lipid **8 p**/chol mixture. However, the diameter of a double‐stranded DNA is ∼20 Å.^63^ Comparing these two *d* values leads to the conclusion that the incorporation of DNA induces a partial squeeze‐out of water between the lipid bilayers or a changed orientation of the lipid head groups connected with changes in the tilt angle of the chains.^66^ The absence of any other diffraction peak in the SAXS pattern proves that the lipoplex forms a homogenous gel phase, in contrast to lipid **8**/chol/DNA, which shows the coexistence of two lamellar phases: one with incorporated DNA (s_001_=0.0117 Å^−1^, *d*=85.5 Å) and one without DNA (s_001_=0.0133 Å^−1^, *d*=75.2 Å) (Figure 9).^30^

Heating the sample leads to the main phase transition at ∼40 °C with the appearance of a lamellar phase with a *d*‐value of 73 Å. This value is only 8 Å larger compared to the L_α_ phase in the lipid **8 p**/chol mixture without DNA. Even if the increase in *d* is quite small, this liquid‐crystalline phase should contain DNA. Further heating leads to another Bragg peak at the position expected for the pure L_α_ phase (*d* ∼65 Å) with no DNA incorporated. It is very interesting to note that the appearance of the Q_α_ phase is completely suppressed by the incorporation of DNA. Cooling the sample from 50 °C to 20 °C shows that the L_α_ phase containing the DNA can be markedly super‐cooled (Figure S16). Additionally, it is interesting to compare the DSC cooling curves of lipid **8 p**/chol mixture without DNA (Figure S15) and with DNA (Figure S17). After adding the DNA, the small transition attributed to Q_α_–L_α_ is not present in accordance with the X‐ray results showing the absence of the Q_α_ phase in the DNA containing mixture.

### 2.4 Transfection Experiments

**Figure 10:**
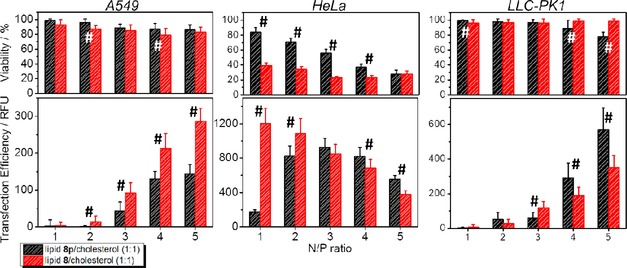
Determination of transfection efficiencies in A549, HeLa, and LLC‐PK1 cells by eGFP assay (lower panels) and cell viability by alamarBlue assay (upper panels) of 1 : 1 mixtures of the cationic lipid with cholesterol at different N/P ratios with pDNA after 24 h. The hashtag (**#**) indicates statistical significant differences between the formulations bearing lipid **8 p** or lipid **8** using the OneWay ANOVA test with an α of 0.05 (n=12). Detailed information of the OneWay ANOVA test can be found in the Supporting Information.

Figure 10 shows the transfection efficiencies and the cell viability after treatment of three different cell lines with lipoplexes composed of lipid **8 p**/chol (1 : 1) or lipid **8**/chol (1 : 1) at different N/P ratios. Commonly used standard cell lines for screening of transfection efficacy were used: A549 (human lung epithelial cells), HeLa (human cervix epithelial cells) and LLC‐PK1 (porcine kidney epithelial cells). Apart from a few exceptions, both lipid composites show comparable transfection properties. The transfection efficiency values show significant differences in most N/P ratios in all 3 cell lines, but in most cases the difference is not very pronounced (see also F and P values of the OneWay ANOVA in the Supporting Information Table S3) for a biological system and there is no general trend which lipid is more beneficial. In A549 cells, the mixture with lipid **8** is slightly better. At N/P 5, the difference becomes highly significant. In HeLa cells, the composite with lipid **8** is significantly more effective at N/P 1 and slightly more effective at N/P 2 whereas the composite with lipid **8 p** is slightly more effective at the N/P 4 and N/P 5. At N/P 3 no significant difference exists for the transfection efficiency. In LLC‐PK1 cells, the mixture with lipid **8 p** exhibits slightly higher transfection efficiencies. However, the viability of the used cells after treatment with the lipoplexes is almost the same for A549 cells and LLC‐PK1 cells with no significant differences at most evaluated N/P ratios, but rather different for HeLa cells. Here lipid **8** has higher cytotoxic effects at lower N/P ratios. At N/P 5, the cytotoxic behavior of the composite containing lipid **8** is comparable to the composite with lipid **8 p**.

## 3 Discussion and Conclusion

The main question to be answered by the present results is whether oleyl derivatives of only technical purity can be successfully used to synthesize cationic lipids as polynucleotide carriers? The extensive physical‐chemical studies and transfection experiments of two cationic lipids (lipid **8 p** and lipid **8**) alone and in mixture with cholesterol give the clear answer ‘yes’. The alkyl chain composition i) influences the miscibility behavior (de‐mixing is observed in monolayer and bilayer experiments), ii) has no effect on the DNA binding capacity, and iii) has no relevant effect on the transfection efficiency.

The use of oleylamine of technical grade allows a faster and more cost‐efficient synthesis of the cationic lipid compared with the synthesis of the alkyl chain pure derivative, whereupon the effect on the transfection behavior is marginal. The study clearly shows effects of the alkyl chain purity on the self‐assembly behavior in 2D and 3D systems, but the differences seem not to affect the biological activity of the used lipoplexes. Lipid **8 p** exhibits a defined phase transition (LE→LC) in Langmuir monolayers at pH 10, whereas lipid **8** is characterized by a coexistence of LE and LC phases (IRRAS, BAM). Furthermore, GIXD measurements determined lipid demixing in lipid **8** (oleyl chains of technical grade) by detecting two different alkyl chain lattices. Nevertheless, the DNA binding capacity of both lipids at the air water interface is comparable (IRRAS). In 3D systems, lipid **8 p** forms cubic phases, often discussed as beneficial for effective lipofection) in dependence of the protonation state. Lipid **8** shows no cubic phases under the same experimental conditions. The cubic phase is also formed in mixtures of lipid **8 p** with cholesterol. However, this cubic phase disappears after addition of DNA. DNA has the ability to suppress the cubic phase also at high temperatures. At physiological temperatures for medical applications in human cells, both lipids (alone and in mixture with cholesterol) assemble to lamellar aggregates. This can explain the comparable biological activity as DNA carriers. Both lipids, lipid **8** and lipid **8 p**, form lamellar assemblies in mixtures with cholesterol after complex formation with DNA. The only difference is the amount of incorporated DNA. Lipoplexes using lipid **8** segregate into a DNA‐free and a DNA‐containing lamellar phase, whereas lipoplexes with lipid **8 p** are homogeneous one‐phase systems.

## Supporting Information

Details of lipid synthesis and analysis of lipid purity; monolayers: pressure/area isotherms and IRRAS at different temperatures, table of parameters derived from GIXD; bulk: table of thermodynamic parameters derived from DSC, additional DSC scans, SAXS and WAXS temperature scans, and additional information to the statistical analysis.

## Conflict of interest

The authors declare no conflict of interest.

## Supporting information

As a service to our authors and readers, this journal provides supporting information supplied by the authors. Such materials are peer reviewed and may be re‐organized for online delivery, but are not copy‐edited or typeset. Technical support issues arising from supporting information (other than missing files) should be addressed to the authors.

Supplementary
