# Supplementary material for: The Impact of Alkyl‐Chain Purity on Lipid‐Based Nucleic Acid Delivery Systems – Is the Utilization of Lipid Components with Technical Grade Justified?
Source: Chemphyschem. 2019 Jul 23;20(16):2110–21. doi: 10.1002/cphc.201900480 (PMC6771585; doi:10.1002/cphc.201900480)
Supplement: Supplementary file 1 — Supplementary [file CPHC-20-2110-s001.pdf]

CHEMPHYSCHEM

Supporting Information

© Copyright Wiley-VCH Verlag GmbH & Co. KGaA, 69451 Weinheim, 2019

# The Impact of Alkyl-Chain Purity on Lipid-Based Nucleic Acid Delivery Systems – Is the Utilization of Lipid Components with Technical Grade Justified?

Dorota Pawlowska, Christopher Janich, Andreas Langner, Bodo Dobner, Christian Wölk,* and Gerald Brezesinski©2019 The Authors. Published by Wiley-VCH Verlag GmbH & Co. KGaA. This is an open access article under the terms of the Creative Commons Attribution License, which permits use, distribution and reproduction in any medium, provided the original work is properly cited.

**Supporting information**

# The impact of alkyl chain purity on lipid based nucleic acid delivery systems – is the utilization of lipid components with technical grade justified?

*Dorota Pawlowska,*[a,b] *Christopher Janich,*[c] *Andreas Langner,*[c] *Bodo Dobner,*[c] *Christian Wölk,*[c*] *and Gerald Brezesinski* [a]

[a]Max Planck Institute of Colloids and Interfaces, Science Park Potsdam-Golm, Am Mühlenberg 1, 14476 Potsdam, Germany. Fax: +493315679202; Tel: +493315679234; E-mail: brezesinski@mpikg.mpg.de

[b]Warsaw University of Technology, Faculty of Chemistry, Institute of Biotechnology, ul. Noakowskiego 3, 00-664 Warsaw, Poland. Fax: +48226282741; Tel: +48222345734;

[c]Martin Luther University, Institute of Pharmacy, Research Group Biochemical Pharmacy, Wolfgang-Langenbeck-Str. 4, Halle (Saale), Germany. Fax: +49 (345) 5527018; Tel: +493455525120; E-mail: christian.woelk@pharmazie.uni-halle.de

**Content**


**S1.** **Scheme and instructions for synthesis of lipid 8p**

**S2.** **Analytical data of pure oleylamine, lipid precursor-compounds bearing the pure oleylamine and lipid 8p**

**S3.** **NMR-spectra of lipid 8p**

**S4.** **Biological experiments**

**S5.** **Isotherms and IRRAS of lipid 8p and lipid 8**

**S6.** **Thermodynamics of lipid 8p phase transition**

**S7.** **Brewster Angle Microscopy (BAM) of lipid 8**

**S8.** **GIXD of lipid 8p**

**S9.** **Example IRRA spectra**

**S10.** **DSC – thermodynamic parameters of lipid 8p and lipid 8**

**S11.** **SAXS/WAXS (temperature scans) and DSC of lipid 8p, lipid 8p/cholesterol and lipid 8p/cholesterol/DNA**

**S12.** **Parameter of the OneWay ANOVA performed with the cell culture assays**

**S1. Scheme and instructions for synthesis of lipid 8p**

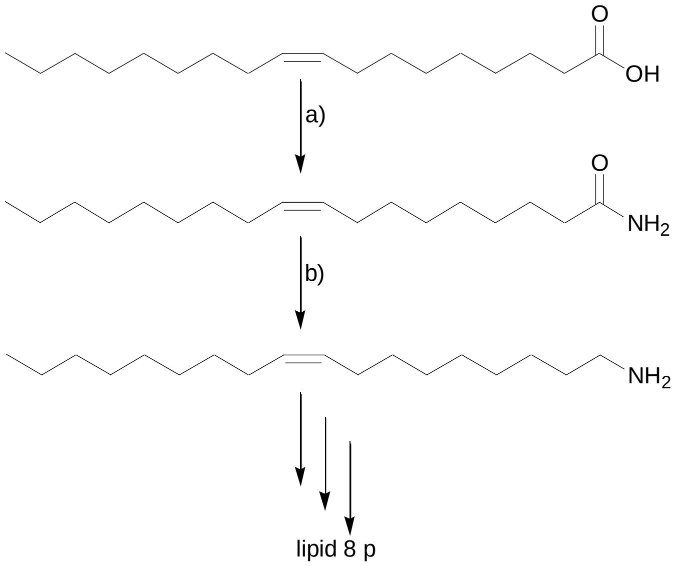


**Fig. S1** Scheme of synthesis of oleylamine pure with oleic acid as educt and oleic acid amide as intermediate product: a) 1. ethyl chloroformate, TEA, THF 2. $NH_3$, THF; b) 1. $LiAlH_4$, THF 2. $H_2O$. The oleylamine pure was used for the synthesis of lipid **8p**.

**Synthesis of pure oleylamine**. Pure oleic acid (0.018 mol, 5 g) and TEA (0.018 mol, 2.5 mL) were dissolved in THF (20 mL). After the solution was cooled down to -5 °C, ethyl chloroformate (0.018 mol, 1.95 g), dissolved in THF (20 mL), was added dropwise within 30 min, and the mixture was stirred for 1 h obtaining the activated carboxylic acid. Ammonia was blown into THF (20 mL) at -40 °C for 5 min, and the activated carboxylic acid was dropwise added. The mixture was allowed to reach 0 °C and was stirred at this temperature for 5 h. Following, the mixture was evaporated to the half of the original volume and extracted three times with ether (40 mL). The organic layers were collected, washed with brine, dried over sodium sulfate, filtered, and the solvent was evaporated. The crude product was dissolved in THF (30 mL) and then dropped into slurry of lithium aluminum hydride (0.025 mol, 0.95 g) in THF (40 mL). The mixture was heated to 55 °C for 8 h followed by quenching at 0 °C with water and addition of saturated ammonium chloride solution. The

mixture was extracted two times with ether (50 mL). The organic layers were combined, washed with brine, dried over sodium sulfate, filtered, and the solvent was evaporated. The crude oleylamine was purified by column chromatography using $CHCl_3$/MeOH/$NH_3$ and gradient technique.

The lipid **8p** and lipid **8** were synthesized as published earlier.[1] The synthesis was modified by replacing EEDQ by PyBOP™ because of the higher yields and a shorter reaction time. We used oleylamine tech. purchased from Sigma-Aldrich for the synthesis of lipid 8, and oleylamine pure (synthesis described above) for the synthesis of lipid 8p. The lipid 8 and the precursor-compounds were characterized earlier.[1]

## S2. Analytical data of pure oleylamine, lipid precursor-compounds bearing the pure oleylamine and lipid 8p.

***Oleylamine pure($C_{18}H_{37}N$, MW = 267.49 g/mol).*** Colourless liquid, yield = 39 %. $R_f$: 0.67 ($CHCl_3$/MeOH/$NH_3$; 80/20/2; v/v/v). ESI-MS: 268.2 $(M+H)^+$. $^1$H NMR ($CDCl_3$, 400 MHz) δ (ppm): 0.85 (t, $J$ = 6.8 Hz, 3H; $C\underline{H}_3$), 1.26-1.29 (m, 22H; alkyl), 1.40-1.45 (m, 2H; $C\underline{H_2}CH_2NH_2$), 1.98-2.05 (m, 4H; $C\underline{H_2}CH{=}CH\underline{CH_2}$), 2.67 (t, $J$ = 7.1 Hz, 2H; $C\underline{H_2}NH_2$), 5.30-5.38 (m, 2H; $C\underline{H}{=}C\underline{H}$). $^{13}$C NMR ($CDCl_3$, 100 MHz) δ (ppm): 14.0 ($\underline{C}H_3$), 22.6 ($\underline{C}H_2CH_3$), 26.8, 27.11, 27.13, 29.17, 29.24, 29.38, 29.44, 29.67, 31.8 ($\underline{C}H_2CH_2CH_3$), 33.3 ($\underline{C}H_2CH_2NH_2$), 42.0 ($\underline{C}H_2NH_2$), 129.7 129.8 ($\underline{C}H{=}\underline{C}H$).

## GC-MS-spectra of Oleylamine pure and tech.

Area Percent Report -- Sorted by Signal

Information from Data File:
File : C:\HPCHEM\1\DATA\1\WOELK1.D
Operator : E.L.
Acquired : 27 Jan 109 2:30 pm using AcqMethod SCAN1
Sample Name: OA M
Misc Info : 2,7/1-70/10-250
Vial Number: 1
CurrentMeth: C:\HPCHEM\1\METHODS\DEFAULT.M

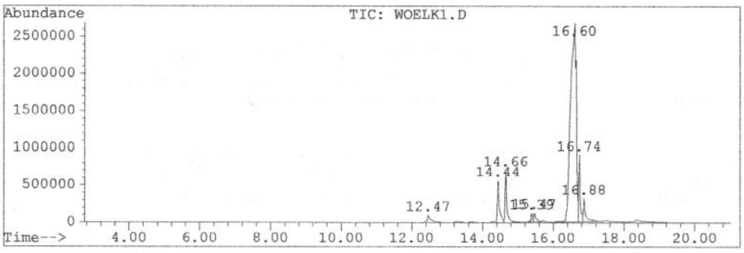


| Retention Time | Area | Area % | Ratio % |
|---|---|---|---|
| Total Ion Chromatogram | | | |
| 12.470 | 6834622 | 1.665 | 2.218 |
| 14.443 | 22694648 | 5.528 | 7.365 |
| 14.662 | 24307230 | 5.921 | 7.889 |
| 15.394 | 3203374 | 0.780 | 1.040 |
| 15.468 | 6029046 | 1.469 | 1.957 |
| 16.604 | 308126259 | 75.058 | 100.000 |
| 16.740 | 25274802 | 6.157 | 8.203 |
| 16.876 | 14046676 | 3.422 | 4.559 |

C:\HPCHEM\1\DATA\1\WOELK1.D Wed Jan 28 06:21:30 2009 Page 1

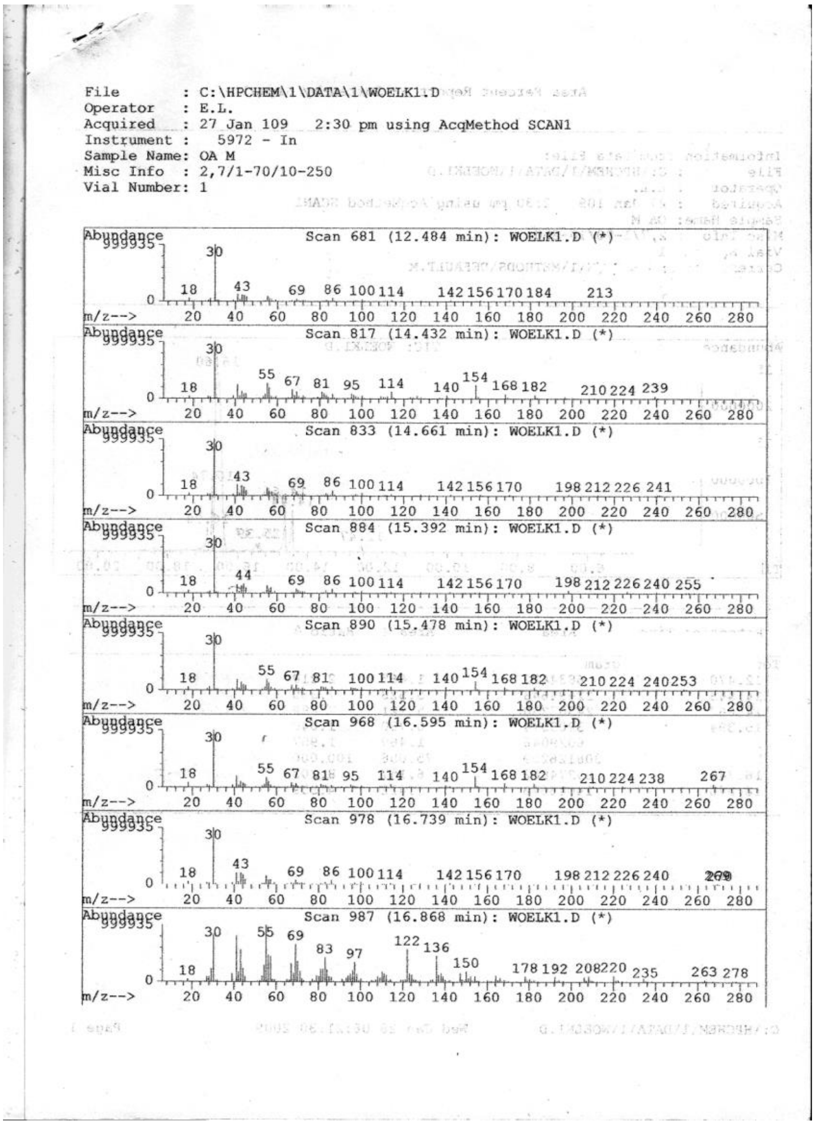


**Fig. S2** GC-MS spectra of oleylamine of technical grade.

Area Percent Report -- Sorted by Signal

Information from Data File:
File        : C:\HPCHEM\1\DATA\1\WOLK6.D
Operator    : Schwarzer
Acquired    : 15 Jan 114    7:08 am using AcqMethod SCHWA70
Sample Name: C18H37N
Misc Info   : 2,5/1-70/10-250
Vial Number: 1
CurrentMeth: C:\HPCHEM\1\METHODS\DEFAULT.M

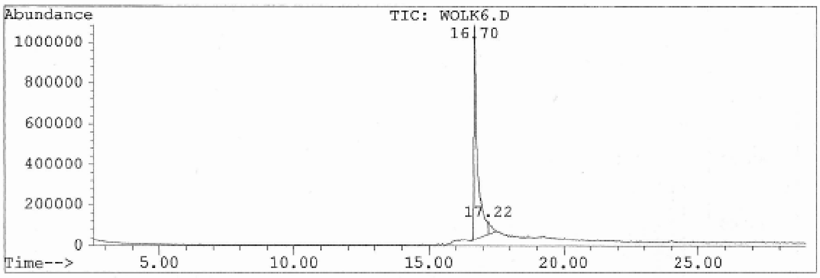


| Retention Time | Area | Area % | Ratio % |
|---|---|---|---|
| Total Ion Chromatogram | | | |
| 16.703 | 91454672 | 94.712 | 100.000 |
| 17.223 | 5106240 | 5.288 | 5.583 |

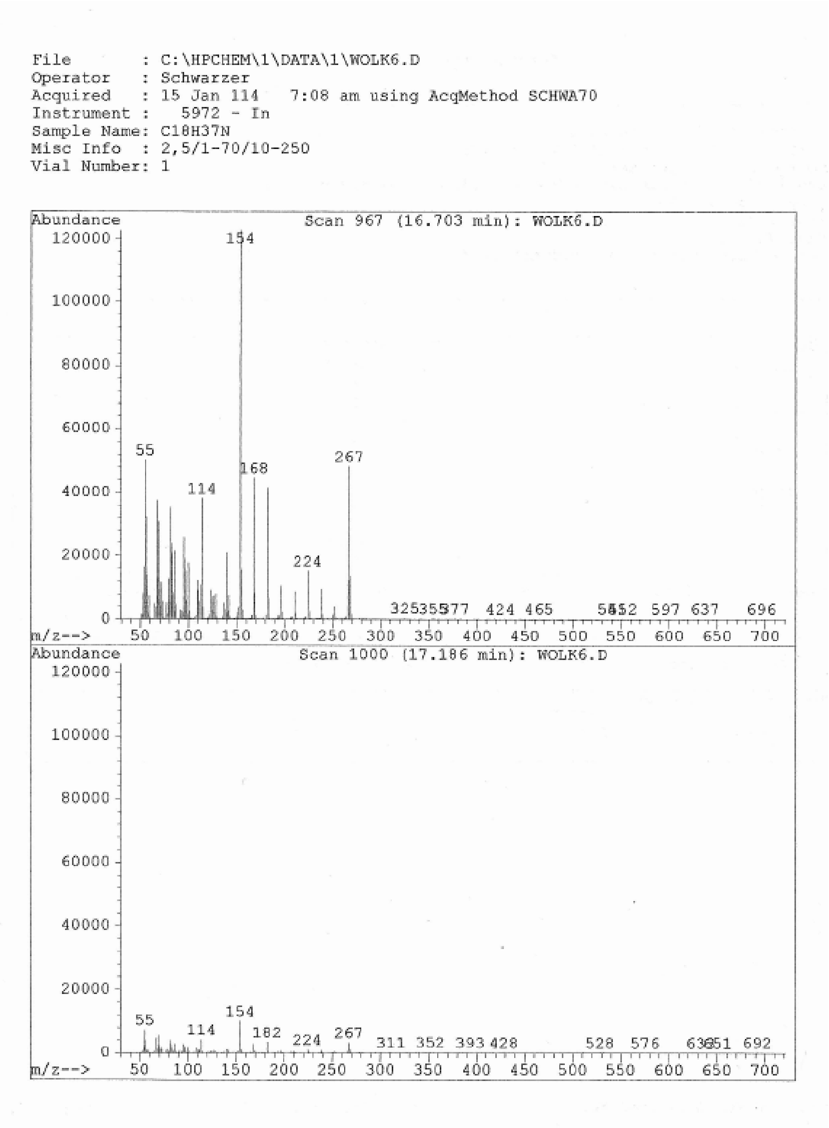


**Fig. S3** GC-MS spectra of pure oleylamine.

***2-{[(9Z)-Octadec-9-enylamino]carbonyl}octadecanoic acid ethyl ester pure ($C_{39}H_{75}N_1O_3$, M = 606.03)***

White waxy solid. $R_f$: 0.28 ($CHCl_3$/heptane; 6/4; v/v). ESI-MS: 576.3 $(M+H)^+$. m.p 48-50°C. $^1$H NMR ($CDCl_3$, 500 MHz) δ (ppm): 0.88 (t, *J* = 6.3 Hz, 6H; 2×$C\underline{H}_3$), 1.25-1.39 (m, 52H; alkyl), 1.49 (t, *J* = 6.1 Hz, 3H; $OCH_2C\underline{H}_3$), 1.86-1.88 (m, 2H; $C\underline{H}_2CH(CONH)_2$), 2.00-2.02 (m, 4H; $C\underline{H}_2CH{=}CH\underline{CH}_2$), 3.16-3.32 (m, 3H; $COC\underline{H}CO$, $C\underline{H}_2NHCO$), 4.16-4.21 (q, *J* = 7.0 Hz, 2H; $OC\underline{H}_2CH_3$), 5.30-5.38 (m, 2H; $C\underline{H}{=}C\underline{H}$), 6.55 (s, 1H; $CH_2N\underline{H}CO$).

***2-{[(9Z)-Octadec-9-enylamino]carbonyl}octadecanoic acid pure ($C_{37}H_{71}N_1O_3$, M = 577.97)***

White waxy solid. $R_f$: 0.61 ($CHCl_3$/MeOH; 8/2; v/v). ESI-MS: 576.3 $(M+H)^+$. m.p 63-69°C. $^1$H NMR ($CDCl_3$, 500 MHz) δ (ppm): 0.88 (t, *J* = 6.8 Hz, 6H; 2×$C\underline{H}_3$), 1.25-1.29 (m, 50H; alkyl), 1.51-1.55 (m, 2H; $CH_2C\underline{H}_2CH_2NHCO$), 1.85-1.97 (m, 2H; $C\underline{H}_2CH(CONH)_2$), 1.99-2.02 (m, 4H; $C\underline{H}_2CH{=}CH\underline{CH}_2$), 3.17 (t, *J* = 6.9 Hz, 1H; $COC\underline{H}CO$), 3.23-3.36 (m, 2H; $C\underline{H}_2NHCO$), 5.32-5.37 (m, 2H; $C\underline{H}{=}C\underline{H}$), 6.26-6.31 (t, *J* = 5.4 Hz, 1H; $CH_2N\underline{H}CO$).

***N-(2-Aminoethyl)-2-hexadecyl-N'-[(9Z)-octadec-9-enyl]propane diamide pure ($C_{39}H_{77}N_3O_2$, M = 620.05)***

Whyte waxy solid. $R_f$: 0.72 ($CHCl_3$/MeOH/$NH_3$; 80/20/2; v/v/v). ESI-MS: 620.4 $(M+H)^+$. m.p 67-70°C. $^1$H NMR ($CDCl_3$, 500 MHz) δ (ppm): 0.88 (t, *J* = 6.8 Hz, 6H; 2×$C\underline{H}_3$), 1.24-1.28 (m, 50H; alkyl), 1.47-1.51 (m, 2H; $CH_2C\underline{H}_2CH_2NHCO$), 1.82-1.87 (m, 2H; $C\underline{H}_2CH(CONH)_2$) 1.96-2.03 (m, 4H; $C\underline{H}_2CH{=}CH\underline{CH}_2$), 2.84 (t, *J* = 5.9 Hz, 2H; $C\underline{H}_2NH_2$), 2.94 (t, *J* = 7.4 Hz, 1H; $COC\underline{H}CO$), 3.20-3.35 (m, 4H; 2×$C\underline{H}_2NHCO$), 5.30-5.39 (m, 2H; $C\underline{H}{=}C\underline{H}$), 6.61 (t, *J* = 5.4 Hz, 1H; $CH_2N\underline{H}CO$), 6.98 (t, *J* = 5.2 Hz, 1H; $CH_2N\underline{H}CO$).

***Lipid 8p ($C_{45}H_{89}N_5O_3$, MW = 748.22 g/mol).*** Colourless crystalline solid. $R_f$: 0.41 ($CHCl_3$/MeOH/$NH_3$; 80/20/2; v/v/v). ESI-MS: 748.4 $(M+H)^+$. HRMS: calc. for $C_{45}H_{90}N_5O_3$: 748.7038 found: 748.7031. $^1$H NMR ($CDCl_3$, 500 MHz) δ (ppm): 0.87 (t, *J* = 6.9 Hz, 6H; 2×$C\underline{H}_3$), 1.23-1.81 (m, 60H; alkyl, $(C\underline{H}_2)_3CH_2NH_2$), 1.98-2.02 (m, 4H; $C\underline{H}_2CH{=}CH\underline{CH}_2$),

2.72 (t, $J$ = 6.7 Hz, 2H; $C\underline{H_2}NH_2$), 2.92 (t, $J$ = 7.5 Hz, 1H; $COC\underline{H}CO$), 3.18-3.41 (m, 7H; 3×$C\underline{H_2}NHCO$, H(α)-lysine), 5.30-5.37 (m, 2H; $C\underline{H}=C\underline{H}$), 6.89-6.93/7.53-7.55/7.63-7.64 (3×m, 3H; 3×$CH_2N\underline{H}CO$). $^{13}C$ NMR ($CDCl_3$, 100 MHz) δ (ppm): 14.1 ($\underline{C}H_3$), 22.6, 22.84, 22.86, 26.9, 27.2, 27.6, 27.7, 29.25, 29.30, 29.34, 29.40, 29.42, 29.46, 29.58, 29.60, 29.64, 29.69, 29.75, 31.88, 31.91, 32.66, 32.75, 32.9, 34.7, 34.8, 38.9 39.0 39.6 39.89 ($\underline{C}H_2NHCO$), 41.6 41.7 ($\underline{C}H_2NH_2$), 55.1 ($CO\underline{C}HCO$, C(α)-lysine), 129.7 129.9 ($\underline{C}H=\underline{C}H$), 170.7 171.9 172.0 176.0 176.6 ($NH\underline{C}O$).

**S3. NMR-spectra of lipid 8p:**

Following we show NMR spectra of lipid **8p** in order to demonstrate that the applied chemical reactions did not have an observable influence on the configuration of the cis double bond. For a better visualisation we show the enlarged region of the double bond signals.

A

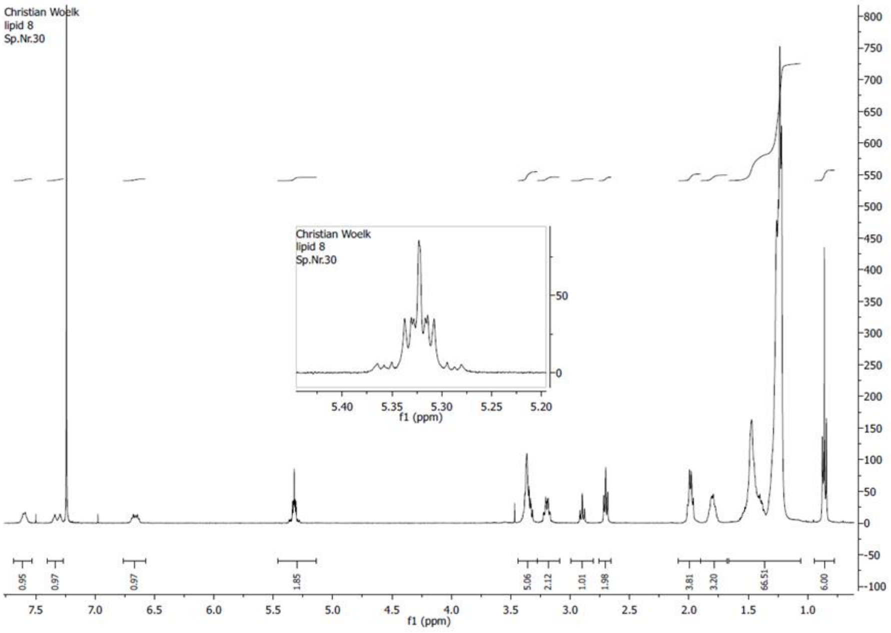

B

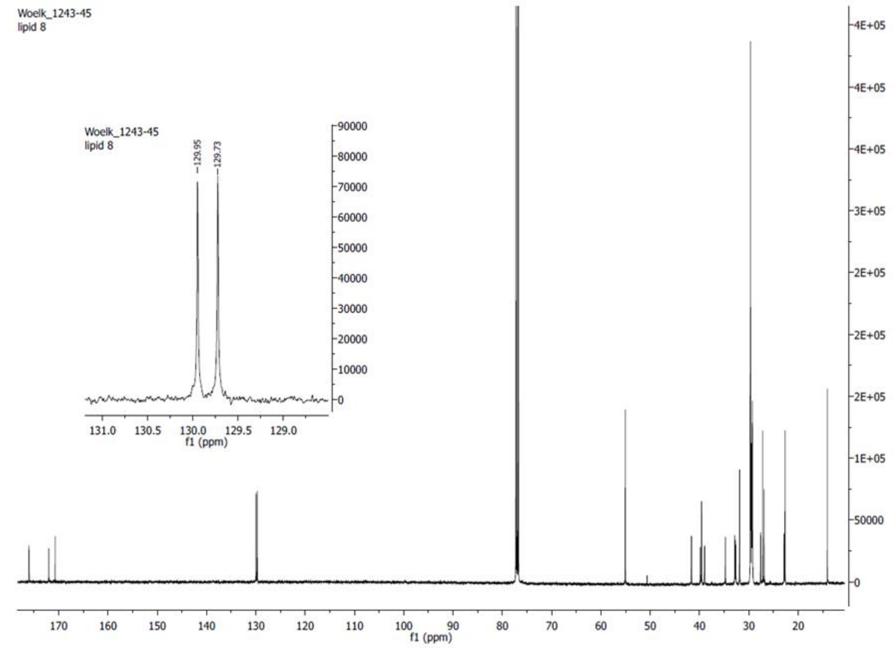


C

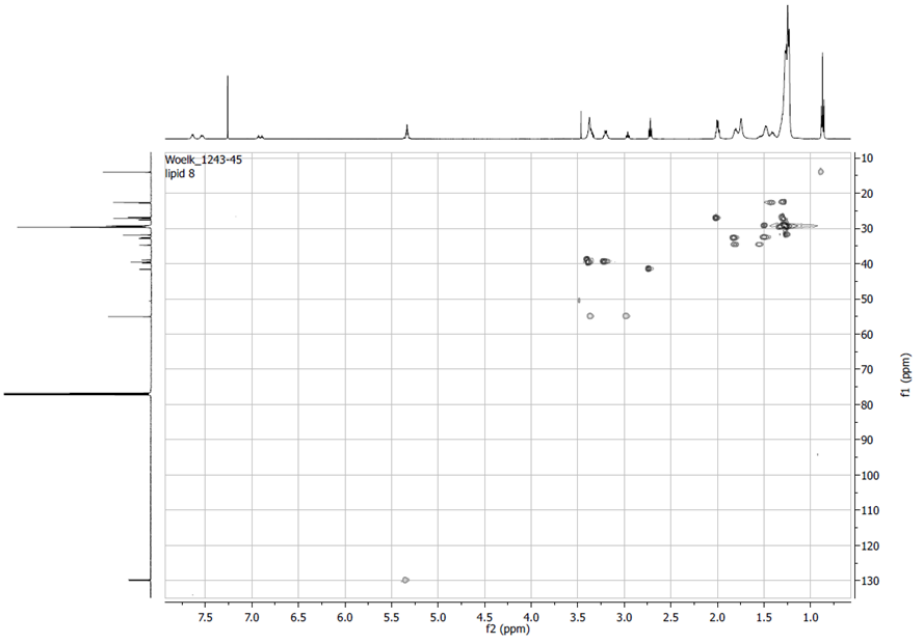


**Fig. S4** $^{1}$H-NMR (A), $^{13}$C-NMR (B) and C,H-COSY (HSQC) (C) spectra of lipid **8p.**

## S4. Biological experiments

**General:** peGFP-C2 plasmid was purchased from Clontech (Mountain View, CA, USA). Plasmid isolation kit was purchased from QIAGEN (Hilden, Germany). A549 cells, HeLa cells and LLC-PK1 cells were aquired from German Collection of Microorganisms and Cell Cultures (DSMZ, Braunschweig, Germany). Cell culture media and fetal bovine serum (FBS) were purchased from Biochrom (Berlin, Germany) and phosphate buffered saline (PBS) was supplied from Sigma Aldrich (Steinheim, Germany). peGFP-C2 was isolated from Escherichia coli DH5α (Invitrogen GmbH, Darmstadt, Germany) using a Quiagen EndoFree® plasmid mega kit following the manufacturer's instructions. The purity of pDNA was verified by the absorbance-quotient $A_{260}/A_{280}$= 2.12 and 1% agarose gel electrophoresis.

***Cell culture.*** A549 cells (human lung carcinoma cells), HeLa cells (human cervix adenocarcinoma epithelial cells), and LLC-PK1 cells (pig kidney epithelial cells) were cultured in 75 $cm^2$ tissue culture flasks in Dulbecco's modified eagle medium (DMEM) adjusted to contain 4.5 $mg \cdot mL^{-1}$ glucose, 10% FBS at 37 °C and 5% $CO_2$. The cells were grown ~90% confluent and were split regularly three times a week. For experiments, cells in the range of passages 10–30 were used.

***Transfection and cell viability assay.*** Cells were seeded into a 96-well plate at a density of $1 \cdot 10^4$–$1.1 \cdot 10^4$ cells/well 24 h before transfection.. Lipoplex mixtures were prepared by combining plasmid DNA with varying amounts of lipid dispersion. The plasmid DNA was added to the lipid dispersion in one step followed by 15 min incubation at 25°C. Cells were washed once with PBS. Then the lipoplexes were added to the cells (0.1 µg DNA per well). 60 µL of DMEM and and FBS were added to the cells (final concentration of FBS reached 10%). 24 h after transfection, the eGFP fluorescence was measured using a BMG 10 filter ($\lambda_{ex}$ = 485 nm, $\lambda_{em}$ = 520 nm) with a fixed gain. Therefore, the cells were washed twice with PBS

and filled up with 100 µL PBS. The transfection efficiency determined as eGFP fluorescence was auto-fluorescence corrected and measured in relative fluorescence units (RFU). For the cytotoxicity assay, the same plates were used and 100 µL of a 2% alamarBlue®/PBS solution (Invitrogen GmbH, Darmstadt, Germany) was added to every well. The plates were incubated for 1 h at 37 °C and 5% $CO_2$. The fluorescence signal was measured using a BMG 10 filter ($\lambda_{ex}$ = 544 nm, $\lambda_{em}$ = 590 nm) with a fixed gain. Viabilities above 100% were set to 100%. All experiments were performed six times and were repeated on two different days (n= 12).

## S5. Isotherms and IRRAS of lipid 8p and lipid 8

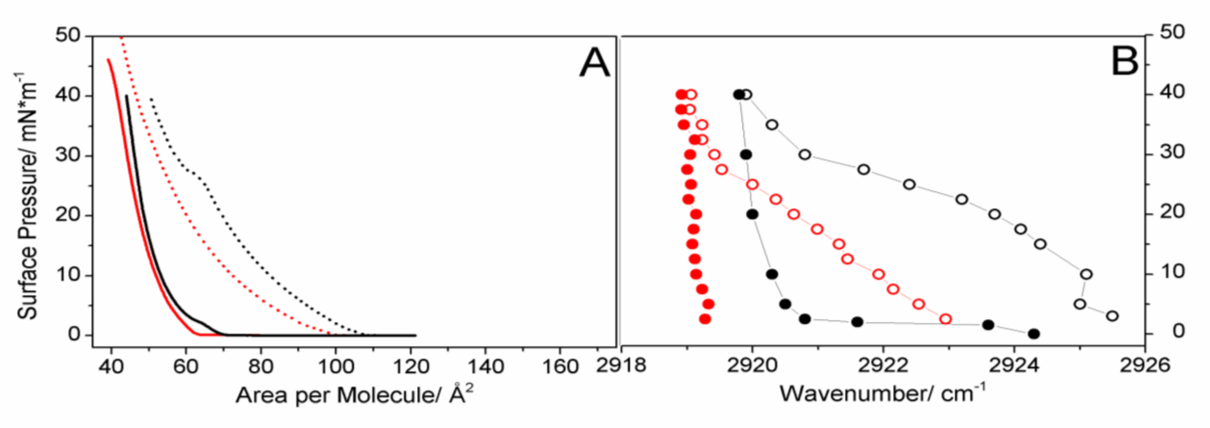


**Fig. S5** Pressure/area isotherms (A) and the positions of $\nu_{as}$ ($CH_2$) band along the compression isotherm at 5 °C (B) of lipid **8p** (black) and lipid **8** (red) on buffers: citric buffer pH 4 (A: dashed line, B: empty circles) and carbonate buffer pH 10 (A: solid line; B: filled circles).

## S6. Thermodynamics of lipid 8p phase transition

***Temperature dependence of pressure-area isotherms.*** Since lipid **8p** shows a well-defined first-order phase transition between LE and LC at pH 10, the isotherms have been measured at different temperatures (Fig. S6). The plateau characterizing the phase transition starts with an overcompression (hump at the beginning of the plateau region). The overcompression increases with increasing temperature. The temperature dependence of the transition pressure $\pi_t$, determined from the kink at the beginning of the plateau region (coexistence of LE and LC phases) but neglecting the overcompression, can be described by a linear function (Figure S6 inlay). Extrapolation to zero transition pressure gives a $T_0$ value of 11.2 °C, which determines the lowest temperature of the existence of the liquid-expanded phase. Below this temperature, the transition into the condensed phase starts directly from the gas-analogous state (re-sublimation). The slope $d\pi_t/dT$ of the linear function is 0.806 mN/(m·K). The two-dimensional Clausius-Clapeyron equation (1) representing a one-component approximation can be used for calculating the enthalpy change $\Delta H$ of the phase transition

$$\Delta H = (A_c - A_e)T\frac{d\pi_t}{dT} \qquad (1)$$

where $A_e$ is the molecular area at the onset of the phase transition at the surface pressure $\pi_t$ and $A_c$ is the area of the condensed phase at this pressure.[1] The temperature dependence of the entropy change $\Delta S = \Delta H/T$ for the phase transition is presented in Figure S6. Negative $\Delta S$ values are obtained according to the exothermic nature of the main phase transition and an increase in the ordering of the system. The absolute $\Delta S$ values decrease as the temperature increases until reaching the critical temperature ($T_c$) of 45.4 °C above which the monolayer cannot be compressed to the condensed state. This value is considerably lower than the main transition temperature in bulk (57.1 °C), indicating that the packing in bilayers must be different compared to that in monolayers.

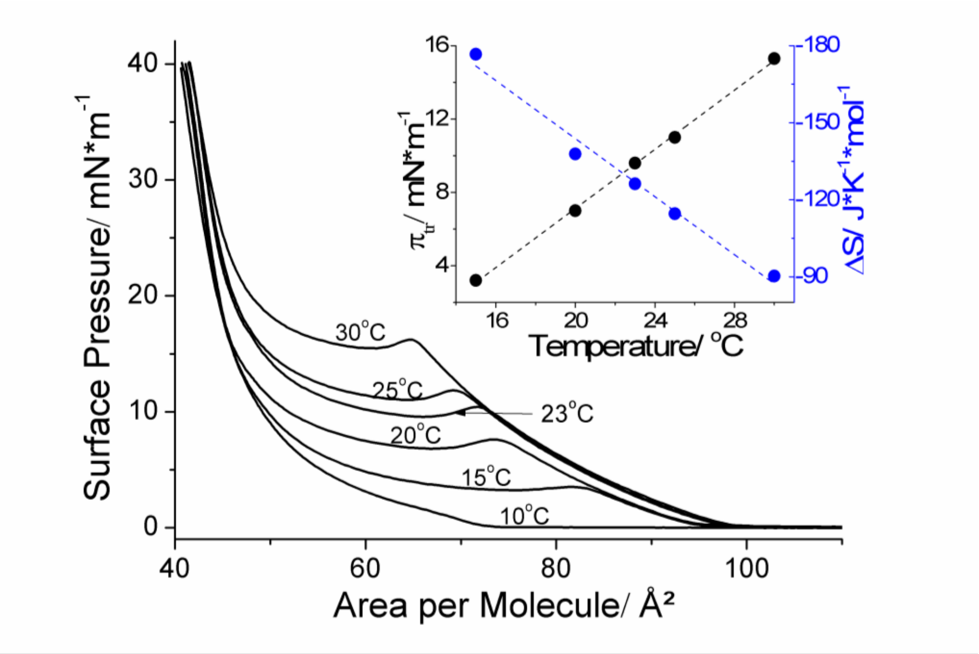


**Fig. S6** Surface pressure/molecular area isotherms of lipid **8p** spread on carbonate buffer, pH 10, at different temperatures (indicated). In the inset the transition pressure (black), $\pi_t$, and transition entropy (blue), ΔS, are plotted in dependence on temperature. The extrapolation of the presented linear fits (dashed lines) yields the characteristic temperatures ($T_0$ = 11.2 °C and $T_c$ = 45.4 °C) described in the text.

The phase transition pressure does not only change in dependence on temperature but also on pH. At lower pH values, the transition pressure of lipid **8p** is shifted to higher values due to increasing electrostatic repulsion between the now charged head groups. At pH 4 and 5 °C, the phase transition starts at $\pi_{tr}$ ~ 25 mN/m (Figure S5). For comparison, the electrostatic repulsion at pH 4 has the same influence on the transition pressure as increasing the temperature to 43.5 °C at pH 10 when the head groups are not charged.

## S7. Brewster Angle Microscopy (BAM) of lipid 8

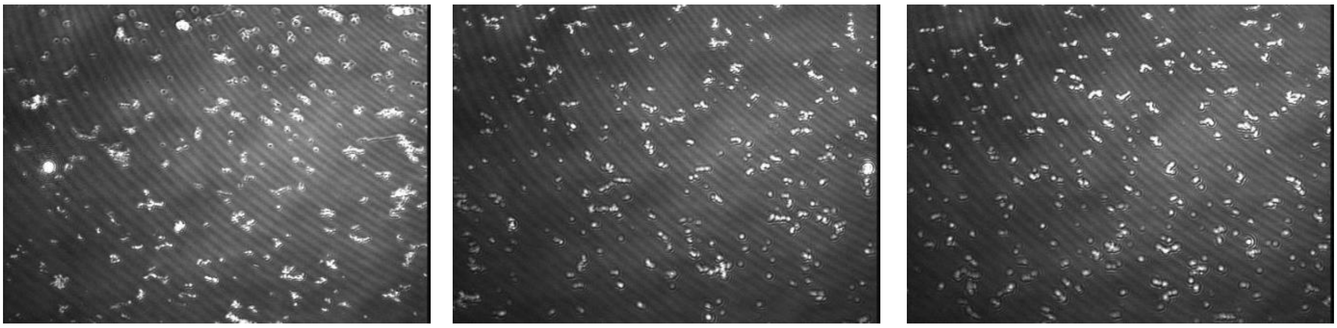

**0.3 mN/m** **0.6 mN/m** **5.0 mN/m**

**Fig. S7** BAM pictures of lipid **8** at pH4, 5mM citrate buffer, at 20°C and different surface pressures.

## S8. GIXD of lipid 8p

**Table S1**. Bragg Peaks ($Q_{xy}$) and Bragg rods ($Q_z$) maxima, corresponding correlation lengths ($L_{xy}$ and $L_z$), tilt angle ($t$), distortion ($d$) and chain cross-sectional area ($A_0$) of lipid **8p** at different surface pressures (π) on buffer, pH 10, and 5 °C. The index d denotes the degenerated and n-d the non-degenerated peaks.

| π<br>mN/m | $Q_{xy}^{d}$<br>Å$^{-1}$ | $L_{xy}^{d}$<br>Å | $Q_{xy}^{nd}$<br>Å$^{-1}$ | $L_{xy}^{nd}$<br>Å | $Q_z^{d}$<br>Å$^{-1}$ | $L_z^{d}$<br>Å | $Q_z^{nd}$<br>Å$^{-1}$ | $L_z^{nd}$<br>Å | t<br>°<br>direction | $d$<br>direction | $A_0$<br>Å$^2$ |
|---|---|---|---|---|---|---|---|---|---|---|---|
| 5 | 1.463 | 430 | 1.447 | 296 | 0.225 | 21 | 0.45 | 21 | 17.3<br>NNN | 0.0146<br>NNN | 20.5 |
| 10 | 1.465 | 415 | 1.455 | 230 | 0.,2235 | 21 | 0.447 | 21 | 17.1<br>NNN | 0.0091<br>NNN | 20.4 |
| 15 | 1.467 | 308 | 1.462 | 328 | 0.219 | 20 | 0.438 | 20 | 16.7<br>NNN | 0.0045<br>NNN | 20.3 |
| 20 | 1.467 | 482 | 1.471 | 339 | 0.200 | 20 | 0.400 | 20 | 15.2<br>NNN | 0.0036<br>NN | 20.4 |
| 30 | 1.471 | 263 | 1.499 | 234 | 0.190 | 20 | 0.380 | 20 | 14.2<br>NNN | 0.0253<br>NN | 20.2 |

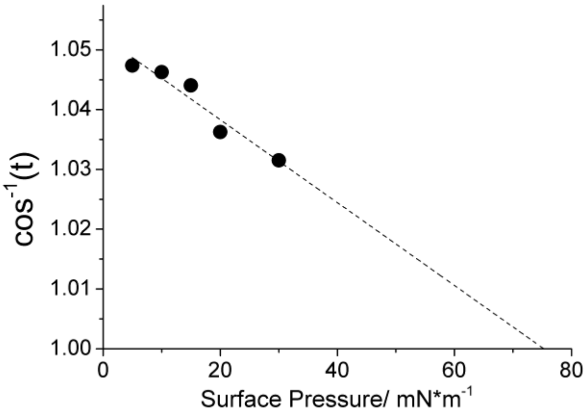


**Fig. S8** Plot of 1/cos(*t)* versus surface pressure for the lipid **8p** monolayer spread on carbonate buffer subphase, pH 10. The extrapolated transition pressure into the non-tilted state of lipid **8p** is $\pi_{tr}$ = 75 mN/m.

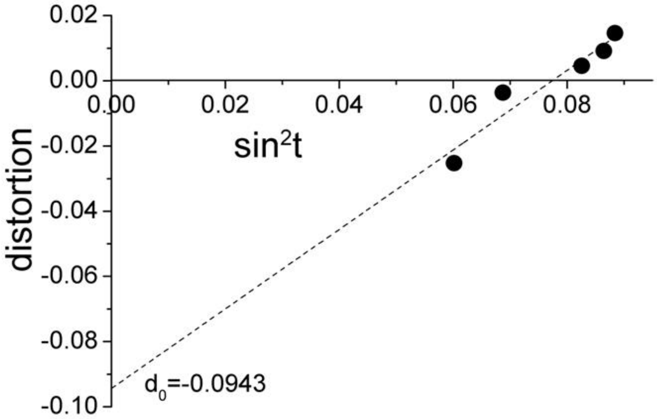


**Fig. S9** Lattice distortion versus $\sin^2(t)$ of the lipid **8p** monolayer spread on carbonate buffer subphase, pH 10.

## S9. Example IRRAS spectra

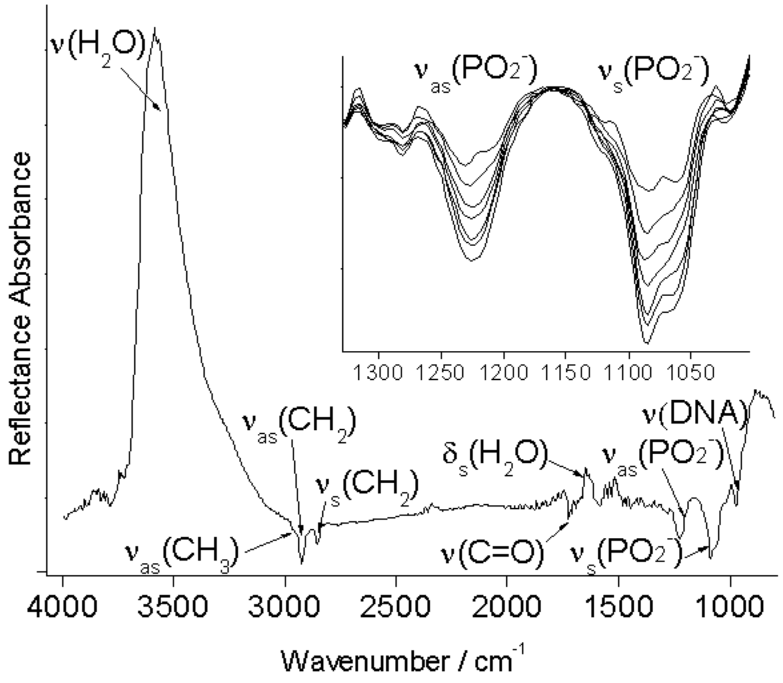


**Fig. S10** IRRA spectra with magnified region of symmetric, $\nu_s$ ($PO_2$), and asymmetric, $\nu_{as}$ ($PO_2$), phosphate diester bands used for the quantification of DNA coupled to the lipid monolayer.

## S10. DSC of lipid 8p and lipid 8

**Table S2.** The main transition temperatures ($T_m$), corresponding transition enthalpies ($\Delta H$), entropies ($\Delta S$) and full-width at half-maximum values of the DSC measurements of the lipids and their mixtures with cholesterol at different pH values. The DSC curves were measured with a scanning rate of 60 $K{\cdot}h^{-1}$. The values were determined from the heating scans.

| | | lipid **8p** | lipid **8** |
|---|---|---|---|
| carbonate buffer, pH 10 | $T_m$ [°C] | 57.1 | 62.1 |
| | $\Delta H$ [kJ $mol^{-1}$] | 41.59 | 30.72 |
| | $\Delta S$ [J $mol^{-1}$ $K^{-1}$] | 125.9 | 91.6 |
| | left half-width [°C] | 1.1 | 6.2 |
| | right half-width [°C] | 0.7 | 2.4 |
| water | $T_m$ [°C] | 53.3 | 54.2 |
| | $\Delta H$ [kJ $mol^{-1}$] | 40.42 | 38.54 |
| | $\Delta S$ [J $mol^{-1}$ $K^{-1}$] | 123.8 | 117,7 |
| | left half width [°C] | 0.7 | 2.7 |
| | right half width [°C] | 0.9 | 1.8 |
| citric buffer, pH 4 | $T_m$ [°C] | 31.1 | 26.0 ($2^{nd}$ peak: 45.2) |
| | $\Delta H$ [kJ $mol^{-1}$] | 37.34 | 16.45 |
| | $\Delta S$ [J $mol^{-1}$ $K^{-1}$] | 122.7 | 54.9 |
| | left half-width [°C] | 3.7 | 5.1 |
| | right half-width [°C] | 2.1 | 7.0 |
| lipid/chol 1/1 water | $T_m$ [°C] | 35.2 | 49.3 |
| | $\Delta H$ [kJ $mol^{-1}$] | 5.42 | 6.44 |
| | $\Delta S$ [J $mol^{-1}$ $K^{-1}$] | 17.5 | 20.0 |
| | left half-width [°C] | 12.5 | 5.8 |
| | right half-width [°C] | 6.1 | 3.2 |

**S11. SAXS/WAXS (temperature scans) and DSC of lipid 8p, lipid 8p/cholesterol and lipid 8p/cholesterol/DNA**

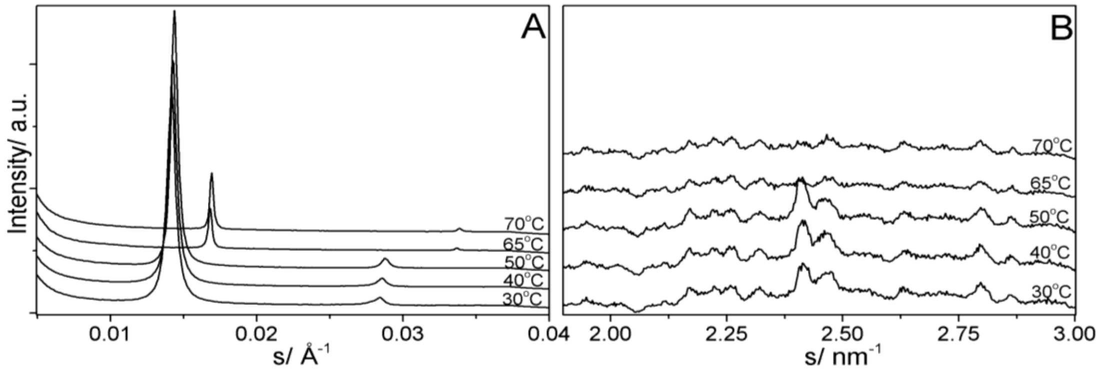


**Fig. S11** SAXS (A) and WAXS (B) of lipid **8p** dispersion (20 wt-% lipid) in carbonate buffer, pH 10, at different temperatures (indicated).

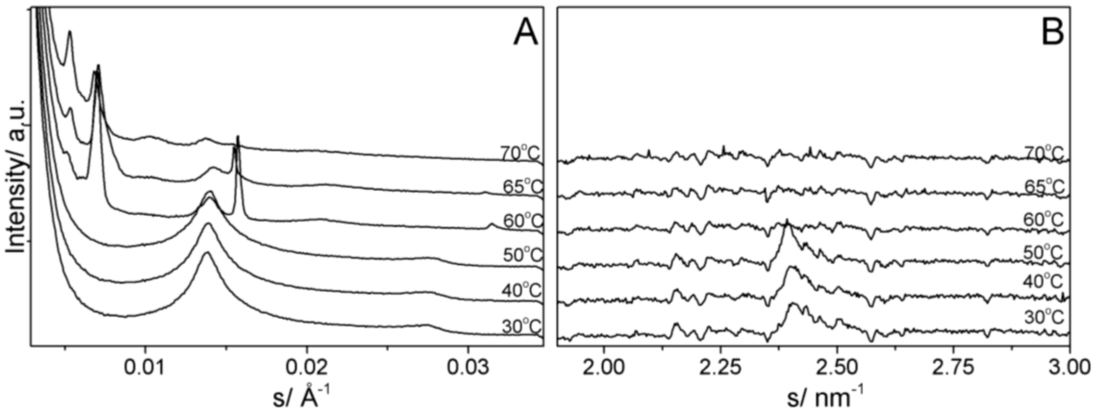


**Fig. S12** SAXS (A) and WAXS (B) of lipid **8p** dispersion (20 wt-% lipid) in water at different temperatures (indicated).

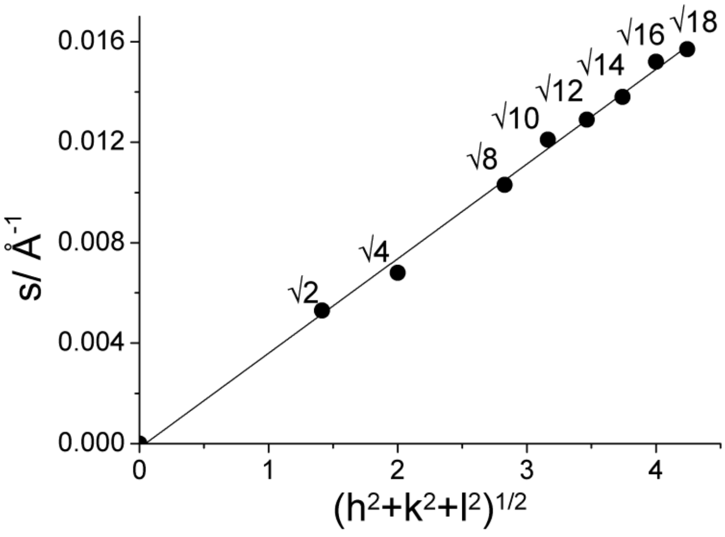


**Fig. S13** Indexed reflexes of body-centered cubic phase of lipid **8p** disspersion in water at 70 °C versus *s* positions.

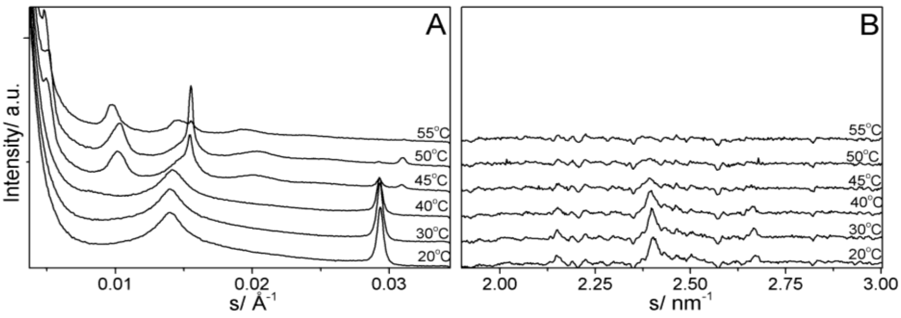


**Fig. S14** SAXS (A) and WAXS (B) of lipid **8p**/chol (1:1) dispersion (20 wt-% mixture) in water at different temperatures (indicated).

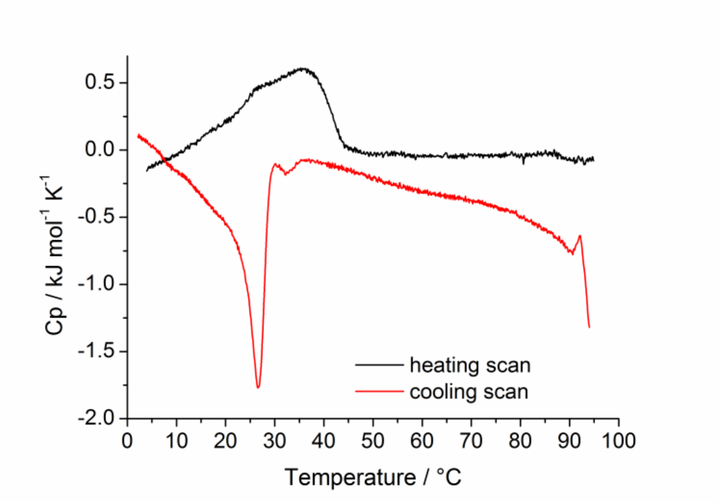


**Fig. S15** DSC heating/cooling scans of lipid **8p**/chol (1:1) dispersion in water.

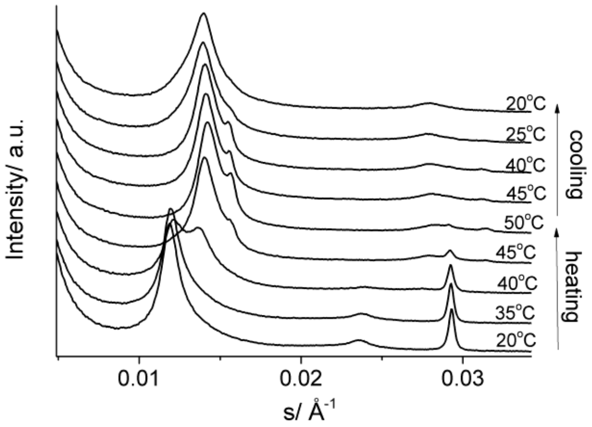


**Fig. S16** Heating and cooling SAXS scans of lipid **8p**/chol (1:1)/ DNA (N/P 3:1) dispersion in water.

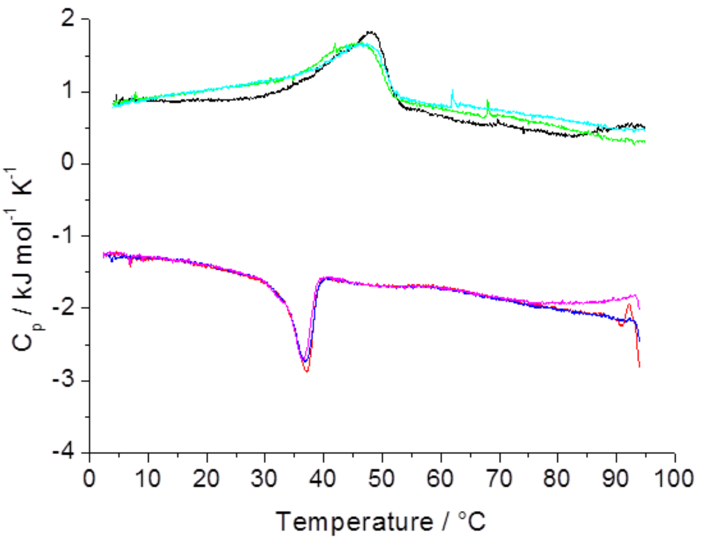


**Fig. S17** Repeated DSC heating/cooling scans of lipid **8p**/chol (1:1)/ DNA (N/P 3:1) dispersion in water.

**S12. Parameter of the OneWay ANOVA performed with the cell culture assays**

**Table S3.** F-value, critical F value and P value of the OneWay ANOVA with an α of 0.05 for the transfection efficiency and viability Tests.

| | N/P ratio | F value | critical F value | P value |
|---|---|---|---|---|
| **A549 cells** | | | | |
| Transfection efficiency | 1 | 0.066073017 | 4.300949502 | 0.799531489 |
| | 2 | 9.41183406 | 4.300949502 | 0.005632112 |
| | 3 | 20.03208302 | 4.300949502 | 0.000188737 |
| | 4 | 39.28777163 | 4.300949502 | 2.61968E-06 |
| | 5 | 131.5877268 | 4.300949502 | 9.39972E-11 |
| viability | 1 | 1.756322643 | 4.300949502 | 0.198683397 |
| | 2 | 19.3805941 | 4.300949502 | 0.,000225795 |
| | 3 | 2.477832412 | 4.300949502 | 0.129733463 |
| | 4 | 6.599323746 | 4.300949502 | 0.017508132 |
| | 5 | 0.86126538 | 4.300949502 | 0.363455588 |
| **HeLa cells** | | | | |
| Transfection efficiency | 1 | 401.1569249 | 4.300949502 | 1.29281E-15 |
| | 2 | 18.57951027 | 4.300949502 | 0.000282676 |
| | 3 | 2.83754593 | 4.300949502 | 0.106220702 |
| | 4 | 11.74436252 | 4.300949502 | 0.00241071 |
| | 5 | 101.7940702 | 4.300949502 | 1.0266E-09 |
| viability | 1 | 0.,05257121 | 4.300949502 | 0.820766266 |
| | 2 | 94.63035355 | 4.300949502 | 1.98971E-09 |
| | 3 | 436.5900277 | 4.300949502 | 5.32773E-16 |
| | 4 | 381.9225251 | 4.300949502 | 2.15919E-15 |
| | 5 | 210.1377264 | 4.300949502 | 7.99157E-10 |
| LLC-PK1 | | | | |
| Transfection efficiency | 1 | 1.065948856 | 4.300949502 | 0.313079354 |
| | 2 | 3.818228567 | 4.300949502 | 0.063527224 |
| | 3 | 18.00600302 | 4.300949502 | 0.000332999 |
| | 4 | 13.02174186 | 4.300949502 | 0.001558962 |
| | 5 | 28.44740465 | 4.300949502 | 2.35791E-05 |
| viability | 1 | 6.179918746 | 4.300949502 | 0.021001353 |
| | 2 | 0.56262787 | 4.300949502 | 0.461150464 |
| | 3 | 0.121804511 | 4.300949502 | 0.730403743 |
| | 4 | 8.637717122 | 4.300949502 | 0.007592944 |
| | 5 | 101.328552 | 4.300949502 | 1.07043E-09 |

1. M. Heinze, G. Brezesinski, B. Dobner and A. Langner, *Bioconjugate Chemistry*, 2010, **21**, 696-708.
